# Perceptual Plasticity for Auditory Object Recognition

**DOI:** 10.3389/fpsyg.2017.00781

**Published:** 2017-05-23

**Authors:** Shannon L. M. Heald, Stephen C. Van Hedger, Howard C. Nusbaum

**Affiliations:** Department of Psychology, The University of Chicago, ChicagoIL, United States

**Keywords:** auditory perception, speech perception, music perception, short-term plasticity, categorization, perceptual constancy, lack of invariance, dynamical systems

## Abstract

In our auditory environment, we rarely experience the exact acoustic waveform twice. This is especially true for communicative signals that have meaning for listeners. In speech and music, the acoustic signal changes as a function of the talker (or instrument), speaking (or playing) rate, and room acoustics, to name a few factors. Yet, despite this acoustic variability, we are able to recognize a sentence or melody as the same across various kinds of acoustic inputs and determine meaning based on listening goals, expectations, context, and experience. The recognition process relates acoustic signals to prior experience despite variability in signal-relevant and signal-irrelevant acoustic properties, some of which could be considered as “noise” in service of a recognition goal. However, some acoustic variability, if systematic, is lawful and can be exploited by listeners to aid in recognition. Perceivable changes in systematic variability can herald a need for listeners to reorganize perception and reorient their attention to more immediately signal-relevant cues. This view is not incorporated currently in many extant theories of auditory perception, which traditionally reduce psychological or neural representations of perceptual objects and the processes that act on them to static entities. While this reduction is likely done for the sake of empirical tractability, such a reduction may seriously distort the perceptual process to be modeled. We argue that perceptual representations, as well as the processes underlying perception, are dynamically determined by an interaction between the uncertainty of the auditory signal and constraints of context. This suggests that the process of auditory recognition is highly context-dependent in that the identity of a given auditory object may be intrinsically tied to its preceding context. To argue for the flexible neural and psychological updating of sound-to-meaning mappings across speech and music, we draw upon examples of perceptual categories that are thought to be highly stable. This framework suggests that the process of auditory recognition cannot be divorced from the short-term context in which an auditory object is presented. Implications for auditory category acquisition and extant models of auditory perception, both cognitive and neural, are discussed.

## Introduction

Perceptual understanding of the auditory world is not a trivial task. We generally perceive discrete auditory objects, despite highly convolved auditory scenes that occur in the real world. For example, we can effortlessly perceive a siren in the distance and the hum of a washing machine while following a dialog in a movie that is underscored by background music. In part, recognizing these sound objects is aided by the spatial separation of the waveforms (see Cherry, 1953) as well as perceptual organization (see Bregman, 1990). However, each of our two basilar membranes is vibrated by the aggregation of the separate source waveforms striking our eardrums. Moreover, each of the sound objects, beyond being mixed in with an uncertain sound stage of other sound objects, may be distorted by the room, by motion, and further may be physically different from the generator of similar objects (washing machine, siren, or talker) we have encountered in the past. Simply stated, there is an incredible amount of variability in our auditory environments.

In speech, the lack of invariance between acoustic waveforms and their intended linguistic meaning became clear when the spectrograph was used to visually represent acoustic patterns in the spectro-temporal domain. Between talkers, there is variation in vocal tract size and shape that translates into differences in the acoustic realization of phonemes (Fant, 1960; Stevens, 1998). However, even local changes over time in linguistic experience (Cooper, 1974; Iverson and Evans, 2007), affective state (Barrett and Paus, 2002), speaking rate (Gay, 1978; Miller and Baer, 1983), and fatigue (Lindblom, 1963; Moon and Lindblom, 1994) can alter the acoustic realization of a given phoneme. Understanding the various sources of variability and their consequences on speech signals is important as different sources of variability may evoke different adaptive mechanisms for their resolution (see, Nygaard et al., 1995).

Beyond sources of variability that seemingly obstruct identification, there is clear evidence that idiosyncratic articulatory differences in how individuals produce phonemes result in acoustic differences (Liberman et al., 1967). Similar sources of variability hold for higher levels of linguistic representation, such as syllabic, lexical, prosodic, and sentential levels of analysis (cf. Heald and Nusbaum, 2014). Moreover, a highly variable acoustic signal is by no means unique to speech. In music, individuals have a perception of melodic stability or preservation of a melodic “Gestalt” despite changes in tempo (Handel, 1993; Monahan, 1993), pitch height or chroma (Handel, 1989), and instrumental timbre (Zhu et al., 2011). In fact, perhaps with a few contrived exceptions (such as listening to the same audio recording with the same speakers in the same room with the same background noise from the same physical location), we are not exposed to the same acoustic pattern of a particular auditory object twice. The question then becomes – how do we perceptually process acoustic variability in order to achieve a sense of experiential stability and recognizability across variable acoustic signals?

## Regularities in our Environment Shape our Perceptual Experience

One possibility is that perceptual stability arises from the ability to form and use categories or classes of functional equivalence. It is a longstanding assertion in cognitive psychology that categorization serves to reduce psychologically irrelevant variability, carving the world up into meaningful parts (Bruner et al., 1956). In audition, some have argued that the categorical nature of speech perception originates in the architecture of the perceptual system (Elman and McClelland, 1986; Holt and Lotto, 2010). Other theories have suggested that speech categories arise out of sensitivity to the statistical distribution of occurrences of speech tokens (for a review, see Feldman et al., 2013).

Indeed, it has been proposed that the ability to extract statistical regularities in one’s environment, which could occur by an unsupervised or implicit process, shapes our perceptual categories in both speech (cf. Strange and Jenkins, 1978; Werker and Tees, 1984; Kuhl et al., 1992; Werker and Polka, 1993; Saffran et al., 1996; Kluender et al., 1998; Maye and Gerken, 2000; Maye et al., 2002) and music (cf. Lynch et al., 1990; Lynch and Eilers, 1991, 1992; Soley and Hannon, 2010; Van Hedger et al., 2016). An often-cited example in speech research is that an infant’s ability to discriminate sounds in their native language increases with linguistic exposure, while the ability to discriminate sounds that are not linguistically functional in their native language decreases (Werker and Tees, 1983). Further, work in speech development by Nittrouer and Miller (1997), Nittrouer and Lowenstein (2007) has shown that the shaping of perceptual sensitivities and acoustic to phonetic mappings by one’s native language experience occurs throughout adolescence, indicating that individuals remain sensitive to the statistical regularities of acoustic cues and how they covary with sound meaning distinctions throughout their development. Therefore, it seems that given enough listening experience, individuals are able to learn how multiple acoustic cues work in concert to denote a particular meaning, even when no single cue is necessary or sufficient.

## Sounds in a System of Categories

Individuals are not only sensitive to the statistical regularities of items that give rise to functional classes or categories, but to the systematic regularities *among* the resulting categories themselves. This hierarchical source of information, which goes beyond any specific individual category, could aid in disambiguating a physical signal that has multiple meanings. For both speech and music this allows the categories within each system to be defined internally, through the relationships held among categories of each system. This suggests that individuals possess categories that work collectively with one another as a long-term, experientially defined context to orchestrate a cohesive perceptual world (see Bruner, 1973; Billman and Knutson, 1996; Goldstone et al., 2012). In music, the implied key of a musical piece organizes the interrelations among pitch classes in a hierarchical structure (Krumhansl and Shepard, 1979; Krumhansl and Kessler, 1982). Importantly, these hierarchical relations become strengthened as a function of listening experience, suggesting that experience with tonal areas or keys shapes how individuals organize pitch classes (cf. Krumhansl and Keil, 1982). These hierarchical relationships are also seen in speech among various phonemic classes, initially described as a featural system (e.g., Chomsky and Halle, 1968) and the distributional constraints on phonemes and phonotactics. For a given talker, vowel categories are often discussed as occupying a vowel space that roughly corresponds to the speaker’s articulatory space (Ladefoged and Broadbent, 1957). Some authors have posited that point vowels, which represent the extremes of the acoustic and articulatory space, may be used to calibrate changes in the space across individuals, as they systematically bound the rest of the vowel inventory (Joos, 1948; Gerstman, 1968; Lieberman et al., 1972). Due to the concomitant experience of visual information and acoustic information (rooted in the physical process of speech sound production), there are also systematic relations that extend between modalities. For example, an auditory /ba/ paired with a visual /ga/ often yields the perceptual experience of /da/ due to the systematic relationship of place of articulation among those functional classes (McGurk and MacDonald, 1976). Given these examples, it is clear that within both speech and music, perceptual categories are not isolated entities. Rather, listening experience over time confers systematicity that can be meaningful. Such relationships may be additionally important to ensure stability in a system that is heavily influenced by recent perceptual experience, as stability may exist through interconnections within the category system. Long-term learning mechanisms may remove short-term changes that are inconsistent with the system, while in other cases, allow for such changes to generalize to the rest of the system in order to achieve consistency.

## Stability of Perceptual Systems?

Despite clear evidence that listeners are able to rapidly learn from the statistical distributions of their acoustic environments, both for the formation of perceptual categories and the relationships that exist among them, few auditory recognition models include such learning^1^. Indeed, speech perception models such as feature-detector theories (e.g., Stevens and Blumstein, 1981), ecological theories (Fowler and Galantucci, 2005), motor theories (e.g., Liberman and Mattingly, 1985), and interactive theories (TRACE: e.g., McClelland and Elman, 1986; C-CuRe: McMurray and Jongman, 2011) provide no mechanism to update perceptual representations, and as such, implicitly assume that the representations that guide the perceptual process are more stable than plastic. While C-CuRE (McMurray and Jongman, 2011) might be thought of as highly adaptive by allowing different levels of abstraction to interact during perception, this model does not make claims about how the representations that guide perception are established either in terms of the formation of auditory objects or the features that comprise them. For example, the identification of a given vowel depends on the first (F1) and second (F2) formant values, but some of these values will be ambiguous depending on the linguistic context and talker. According to C-CuRE, once the talker’s vocal characteristics are known, a listener can make use of these formant values. The listener can compare the formant values of the given signal against the talker’s average F1 and F2, helping to select the likely identification of the vowel. Importantly, for the C-CuRE model, feature meanings are already available to the listener. While there is some suggestion that this knowledge could be derived from linguistic input and may be amended, the model itself has remained agnostic as to how and when this information is obtained and updated by the listener. A similar issue arises in other interactive models of speech perception (e.g., TRACE: McClelland and Elman, 1986; Hebb-Trace: Mirman et al., 2006) and models of pitch perception (e.g., Anantharaman et al., 1993; Gockel et al., 2001).

While some auditory neurobiological models demonstrate clear awareness that mechanisms for learning and adaptation be included in models of perception and recognition (Weinberger, 2004, 2015; McLachlan and Wilson, 2010; Shamma and Fritz, 2014), this is less true for neurobiological models of speech perception, which traditionally limit their modeling to perisylvian language areas (Fitch et al., 1997; Hickok and Poeppel, 2007; Rauschecker and Scott, 2009; Friederici, 2012), ignoring brain regions that have been implicated in category learning, such as the striatum, the thalamus, and the frontoparietal attention-working memory network (McClelland et al., 1995; Ashby and Maddox, 2005). Further, the restriction of speech models to perisylvian language areas marks an extreme cortical myopia of the auditory system, as it ignores the corticofugal pathways that exist between cortical and subcortical regions such as the medial geniculate nucleus in the thalamus, the inferior colliculus in the midbrain, the superior olive and cochlear nucleus in the pons, all the way down to the cochlea in the inner ear (cf. Parvizi, 2009). Previous work has shown that higher-level cognitive functions can reorganize subcortical structures as low as the cochlea. For example, selective attention or discrimination training has been demonstrated to enhance the spectral peaks of evoked otoacoustic emissions produced in the inner ear (Giard et al., 1994; Maison et al., 2001; de Boer and Thornton, 2008). Inclusion of the corticofugal system in neurobiological models of speech would allow the system, through feedback and top-down control, to adapt to ambiguity or change in the speech signal by selectively enhancing the most diagnostic spectral cues for a given talker or expected circumstance, even before it reaches perisylvian language areas. Including the corticofugal system can thus drastically change how extant models, which are entirely cortical, explain top-down, attention modulated effects in speech and music. While the omission of corticofugal pathways and brain regions associated with category learning is likely not an intentional omission but a simplification for the sake of experimental tractability, it is clear that such an omission has large scale consequences for modeling auditory perception, speech or otherwise. Indeed, the inclusion of learning areas and adaptive corticofugal connections on auditory processing requires a vastly different view of perception, in that even the earliest moments of auditory processing are guided by higher cognitive processing via expectations and listening goals. In this sense, it is unlikely that learning and adaptability can be simply grafted on top of current cortical models of perception. The very notion that learning and adaptive connections could be omitted, however, (even for the sake of simplicity) is in essence, a tacit statement that the representations that guide recognition are more stable than plastic.

The notion that our representations are more stable than plastic may also be rooted in our experience of the world as perceptually stable. In music, relative perceptual constancy can be found for a given melody despite changes in key, tempo, or instrument. Similarly, in speech, a given phoneme can be recognized despite changes in phonetic environment and talker. This is not to say that listeners are “deaf” to acoustic differences between different examples of a given melody or phoneme, but that different goals in listening can arguably shape the way we direct attention (consciously or unconsciously) to variability among auditory objects. In this sense, listening goals organize attention, such that individuals orient toward cues that reflect a given parsing, and away from cues that do not (cf. Goldstone and Hendrickson, 2010). More recent work on change deafness demonstrates that changes in listening goals alter a participant’s ability to notice a change in talker over a phone conversation (Fenn et al., 2011). More specifically, the authors demonstrated that participants did not detect a surreptitious change in talker during a phone conversation, but could detect the change if told to explicitly monitor for it. This suggests that listening goals modulate how we parse or categorize signals, in that these listening determine how attention is directed toward the acoustic variance of a given signal.

Perceptual classification or categorization here should not be confused with categorical perception (cf. Holt and Lotto, 2010). *Categorical perception*, classically defined in audition, refers to the notion that a continuum of sounds that differ along a particular acoustic dimension are not heard to change continuously, but rather as an abrupt shift from one category to another (e.g., Liberman et al., 1957). As such, categorical perception suggests that despite changes in listening goals, individuals’ perceptual discrimination of any two stimuli is inextricably linked to the probability of classifying these stimuli as belonging to different categories (e.g., Studdert-Kennedy et al., 1970). Categorization, conversely, refers to a particular organization of attention, wherein cues that are indicative of between-category variability are emphasized while cues that reflect within-category variability are deemphasized (Goldstone, 1994). Indeed, even within the earliest examples of categorical perception (a phenomenon that, in theory, completely attenuates within-category variability), there appears to be some retention of within-category discriminability (e.g., Liberman et al., 1957). English listeners can reliably rate some acoustic realizations of phonetic categories (e.g., “ba”) as better versions than others (e.g., Pisoni and Lazarus, 1974; Pisoni and Tash, 1974; Carney et al., 1977; Iverson and Kuhl, 1995). Additionally, a number of studies have shown that not only are individuals sensitive to within-category variability, but also this variability affects subsequent lexical processing (Dahan et al., 2001; McMurray et al., 2002; Gow et al., 2003). In music, the perception of pitch chroma categories among absolute pitch (AP) possessors is categorical in the sense that AP possessors show sharp identification boundaries between note categories (e.g., Ward and Burns, 1999). However, AP possessors also show reliable within-category differentiation when providing goodness judgments within a note category (e.g., Levitin and Rogers, 2005). Graded evaluations within a category are further seen in musical intervals, where sharp category boundaries indicative of categorical perception are also generally observed at least for musicians (Siegel and Siegel, 1977). There is also evidence that within-category discrimination can exceed what would be predicted from category identification responses (Zatorre and Halpern, 1979). Indeed, Holt et al. (2000) have suggested that the task structure typically employed in categorical perception tasks may be what is driving the manifestation of within category homogeneity that is characteristic of categorical perception. Another way of stating this is that listening goals defined by the task structure modulate the way attention is directed toward acoustic variance.

While there is clear evidence that individuals possess the ability to attend to acoustic variability, even within perceptual categories, it is still unclear from the demonstrations reported thus far whether listeners are influenced by acoustic variability that is attenuated by disattention due to their listening goals. More specifically, it is unclear whether the representations that guide perception are influenced by subtle, within-category acoustic variability, even if it appears to be functionally irrelevant for current listening goals. Even though there is ample evidence that perceptual sensitivity to acoustic variability is attenuated through categorization, this variability may nevertheless be preserved and further, may be incorporated into the representations that guide perception. In this sense, putatively irrelevant acoustic variability, even if not consciously experienced, may still affect subsequent perception. For example, Gureckis and Goldstone (2008) have argued that the preservation of variability (in our case, the acoustic trace independent of the way in which the acoustics relate to an established category structure due to a current listening goal) allows for perceptual plasticity within a system, as adaptability can only be achieved if individuals are sensitive (consciously or unconsciously) to potentially behavioral relevant changes in within-category structure. In this sense, without the preservation of variability listeners would fail to adapt to situations where the identity of perceptual objects rapidly change. Indeed, there is a growing body of evidence supporting the view that the preservation of acoustic variability can be used in service of instantiating a novel category. In speech, adult listeners are able to amend perceptual categories as well as learn novel perceptual categories not present in their native language, even when the acoustic cues needed to learn the novel category structure are in direct conflict with a preexisting category structure. Adult native Japanese listeners, who presumably become insensitive to the acoustic differences between /r/ and /l/ categories through accrued experience listening to Japanese, are nevertheless able to learn this non-native discrimination through explicit perceptual training (Lively et al., 1994; Bradlow et al., 1997; Ingvalson et al., 2012), rapid incidental perceptual learning (Lim and Holt, 2011), as well as through the accrual of time residing in English-speaking countries (Ingvalson et al., 2011). Further, adult English speakers are able to learn the non-native Thai pre-voicing contrast, which functionally splits their native /b/ category (Pisoni et al., 1982) and to distinguish between different Zulu clicks, which make use of completely novel acoustic cues (Best et al., 1988).

Beyond retaining an ability to form non-native perceptual categories in adulthood, there is also clear evidence that individuals are able to update and amend the representations that guide their processing of native speech. Clarke and Luce (2005) showed that within moments of listening to a new speaker, listeners modify their classification of stop consonants to reflect the new speaker’s productions, suggesting that linguistic representations are plastic in that they can be adjusted online to optimize perception. This finding has been replicated in a study that further showed that participants’ lexical decisions reflect recently heard acoustic probability distributions (Clayards et al., 2008).

Perceptual flexibility also can be demonstrated at a higher level, presumably due to discernible higher-order structure. Work in our lab has demonstrated that individuals are able to rapidly learn synthetic speech produced by rule that is defined by poor and often misleading acoustic cues. In this research, no words ever repeat during testing or training, so that the learning of a particular synthesizer is thought to entail the redirection of attention to the most diagnostic and behaviorally relevant acoustic cues across multiple phonemic categories in concert (see Nusbaum and Schwab, 1986; Fenn et al., 2003; Francis et al., 2007; Francis and Nusbaum, 2009) in much the same way as learning new phonetic categories (Francis and Nusbaum, 2002). Given these studies, it appears that the process of categorization in pursuit of current listening goals does not completely attenuate acoustic variability.

Beyond speech, the representations that guide music perception also appear to be remarkably flexible. Wong et al. (2009) have demonstrated that individuals are able to learn multiple musical systems through passive listening exposure. This “bimusicality” is not merely the storage of two, modular systems of music (Wong et al., 2011); though it is unclear whether early exposure (i.e., within a putative critical period) is necessary to develop this knowledge. In support of the notion that even adult listeners can come to understand a novel musical system that may parse pitch space in a conflicting way compared to Western music, Loui and Wessel (2008) have demonstrated that adult listeners of Western music are able to learn a novel artificial musical grammar. In their paradigm, individuals heard melodies composed using the Bohlen–Pierce scale – a musical system that is strikingly different from Western music, as it consists of 13 equally spaced notes within a three-octave range as opposed to 12 equally spaced notes within a two-octave range. Nevertheless, after mere minutes of listening to 15 Bohlen–Pierce melodies that conformed to a finite-state grammar, listeners were able to recognize these previously heard melodies as well as generalize the rules of the finite-state grammar to novel melodies.

Even within the Western musical system, adults display plasticity for learning categories thought to be unlearnable in adulthood. A particularly salient example of adult plasticity within Western music learning comes from the phenomenon of AP – the ability to name or produce any musical note without the aid of a reference note (see Deutsch, 2013 for a review). AP has been conceptualized as a rare ability, manifesting in as few as one in every 10,000 individuals in Western cultures (Bachem, 1955), though the mechanisms of AP acquisition are still debated. While there is some research arguing for a genetic predisposition underlying AP (e.g., Baharloo et al., 1998; Theusch et al., 2009), with even some accounts claiming that AP requires little or no environmental shaping (Ross et al., 2003), most theories of AP acquisition adhere to an early-learning framework (e.g., Crozier, 1997). This framework predicts that only individuals with early note naming experience would be candidates for developing AP categories. As such, previously naive adults should not be able to learn AP. This early-learning argument of AP has been further explained as a “loss” of AP processing without early interventions, either from music or language (i.e., tonal languages), in which AP is emphasized (cf. Sergeant and Roche, 1973; Deutsch et al., 2004). In support of this explanation, infants appear to process pitch both absolutely and relatively, though they switch to relative pitch cues when AP cues become unreliable (Saffran et al., 2005).

Yet, similar to how even “irrelevant” acoustic variability within speech is not completely attenuated, there is mounting evidence that most individuals (regardless of possessing AP) retain the ability to perceive and remember AP, presumably through implicit statistical learning mechanisms. For example, non-AP possessors are able to tell when familiar music recordings have been subtly shifted in pitch (e.g., Terhardt and Seewan, 1983; Schellenberg and Trehub, 2003), even if they are not able to explicitly name the musical notes they are hearing. These results suggest that the perception of AP is not an ability that is completely lost without the knowledge of explicit musical note category labels or with more advanced development of relative pitch abilities. As such, it is possible that adult listeners might be able to learn how musical note categories map onto particular absolute pitches. In support of this idea, most studies examining the degree to which AP can be trained in an adult population find *some* improvement after training, even after a single training session (Van Hedger et al., 2015). A few studies have even found improvements in absolute note identification such that post-training performance rivals that of that an AP population who learned note categories early in life (Brady, 1970; Rush, 1989). These findings not only support the notion that most adults retain an ability to perceive and remember AP to some degree, but also that AP categories are, to an extent, trainable into adulthood.

Despite these accounts of AP plasticity within an adult population, one might still argue that the adult learning of AP categories represents a fundamentally different phenomenon than that of early-acquired AP, even if the behavioral note classifications from trained adults are, in some extreme cases, indistinguishable from that of an AP population who acquired note categories early in life. One reason to support this kind of dissociation between adult-acquired and early-acquired AP relates to the putative lack of plasticity that exists within an AP possessor who acquired note categories early in life. Specifically, note categories within an early-acquired AP population are thought to be highly stable once established (Ward and Burns, 1999), only being alterable in very limited circumstances, such as through physiological changes to the auditory system as a result of aging (cf. Athos et al., 2007) or pharmaceutical interventions (e.g., Kobayashi et al., 2001). However, recent empirical evidence has demonstrated that even within this early-acquired AP population, there exists a great deal of plasticity in note category representations that is tied to particular environmental experiences. Wilson et al. (2012) reported reductions in AP ability as a function of whether an individual plays a “movable *do*” instrument (i.e., an instrument in which a notated “C” actually belongs to a different pitch chroma category, such as “F”), suggesting that nascent AP abilities might be undone through inconsistent sound-to-category mappings. Dohn et al. (2014) reported differences in note identification accuracy among AP possessors that could be explained by whether one was actively playing a musical instrument, suggesting that AP ability might be “tuned up” by recent musical experience.

Both of these studies speak to how particular regularities in the environment may affect overall note category accuracy within an AP population, though they do not speak to whether the *structure* of the note categories can be altered through experience once they are acquired. Indeed, one of the hallmarks of AP is not only being able to accurately label a given pitch with its note category (e.g., C#), but also provide a goodness rating of how well that pitch conforms to the category (e.g., flat, in-tune, or sharp). Presumably, this ability to label some category members as better than others stems from either a fixed note-frequency association established early in life, or through the consistent environmental exposure of listening to music that is tuned to a very specific standard (e.g., in which the “A” above middle C is tuned to 440 Hz). Adopting the first explanation, plasticity of AP category structure should not be possible. Adopting the second explanation, AP category structure should be modifiable and tied to the statistical regularities of hearing particular tunings in the environment. Our previous work has clearly demonstrated evidence in support of this second explanation – that is, the structure of note categories for AP possessors is plastic and dependent on how music is tuned in the current listening environment (Hedger et al., 2013). In our paradigm, AP possessors assigned goodness ratings to isolated musical notes. Not surprisingly, in-tune notes (according to an *A* = 440 Hz standard) were rated as more “in-tune” than notes that deviated from this standard by one-third of a note category. However, after listening to a symphony that was slowly flattened by one-third of a note category, the same participants began rating similarly flattened versions of isolated notes as more “in-tune” than the notes that were in-tune based off of the *A* = 440 Hz standard. These findings suggest that AP note categories are held in place by the recent listening environment, not by a fixed and immutable note-frequency association that is established early in life. Overall, then, the past decade or so of research on AP has highlighted how this ability can be modified by behaviorally relevant environmental input that extends well into adulthood.

## Cross-Domain Transfer Between Music and Speech

These accounts of plasticity in auditory perception for both speech and music suggest that both systems may be subserved by common perceptual and learning mechanisms. Recent work exploring the relationship between speech and music processing has found mounting evidence that musical training improves several aspects of speech processing, though it is debated whether these transfer effects are due to general enhancements in auditory processing (e.g., pitch perception) vs. an enhanced representation of phonological categories. Hypotheses like OPERA (Patel, 2011) posit that musical training may enhance aspects of speech processing when there is anatomical *overlap* between networks that process the acoustic features shared between music and speech, when the perceptual *precision* required of musical training exceed that of general speech processing, when the training of music elicits positive *emotions*, when musical training is *repetitive*, and when the musical training engages *attention*. Indeed, the OPERA hypothesis provides a framework for understanding many of the empirical findings within the music-to-speech transfer literature. Musical training helps individuals to detect speech in noise (Parbery-Clark et al., 2009), presumably through strengthened auditory working memory, which requires directed attention. Musicians are also better able to use non-native tonal contrasts to distinguish word meanings (Wong and Perrachione, 2007), presumably because musical training has made pitch processing more precise. This explanation can further be applied to the empirical findings that musicians are better able to subcortically track the pitch of emotional speech (Strait et al., 2009).

Recent work has further demonstrated that musical training can also influence the categorical perception of speech. Bidelman et al. (2014) found that musicians showed steeper identification functions of vowels that varied along a categorical speech continuum, and moreover these results could be modeled by changes at multiple levels of the auditory pathway (both subcortical and cortical). In a similar study, Wu et al. (2015) found that Chinese musicians were better able to discriminate within-category lexical tone exemplars in a categorical perception task compared to non-musicians, though, unlike Bidelman et al. (2014), the between-category differentiation between musicians and non-musicians was comparable. Wu et al. (2015) interpret the within-category improvement among musicians in an OPERA framework, arguing that musicians have more precise representations of pitch that allow for fine-grained distinctions within a linguistic category.

Finally, there is emerging evidence that certain kinds of speech expertise may enhance musical processing, demonstrating a proof-of-concept of the bidirectionality of music-speech transfer effects. Specifically, non-musician speakers of a tonal language (Cantonese) showed auditory processing advantages in pitch acuity and music perception that non-musician speakers of English did not show (Bidelman et al., 2013). While there is less evidence supporting this direction of transfer, this is perhaps not surprising as speech expertise is ubiquitous in a way music expertise is not. Thus, transfer effects from speech to music processing are more constrained, as one has to design a study in which there (1) exists substantial differences in speech expertise, and (2) this difference in expertise must theoretically relate to some aspect of music processing (e.g., pitch perception).

How can these transfer effects between speech and music be interpreted in the larger context of auditory object plasticity? Given the evidence across speech and music that recent auditory events profoundly influence the perception of auditory objects within each system, it stands to reason that recent auditory experience from one system of knowledge (e.g., music) may influence subsequent auditory perception in the other system (e.g., speech), assuming there is overlap among particular acoustic features of both systems. Indeed, there is some empirical evidence to at least conceptually support this idea. An accumulating body of work has demonstrated that the perception of speech sounds is influenced by the long-term average spectrum (LTAS) of a preceding sound, even if that preceding sound is non-linguistic in nature (e.g., Holt et al., 2000; Holt and Lotto, 2002). This influence of non-linguistic sounds on speech perception appears to reflect a general sensitivity to spectro-temporal distributional information, as the non-linguistic preceding context can influence speech categorization even when it is not immediately preceding the to-be-categorized speech sound (Holt, 2005). While these results do not directly demonstrate that recent experience in music can influence the way in which a speech sound is categorized, it is reasonable to predict that certain kinds of experiences in music or speech (e.g., a melody played in a particular frequency range) may alter the way in which subsequent speech sounds are perceived. As such, future work within this realm will help us understand the extent to which auditory object plasticity can be understood using a general auditory framework.

## Neural Markers for Rapid Auditory Plasticity

What is most remarkable about the previously discussed examples of perceptual plasticity in both speech and music is that significant reorganization of perception can been achieved within a single experimental session. Indeed, there is clear neural evidence from animal models that the ability to rapidly reorganize maps in auditory cortex is maintained into adulthood (see Feldman and Brecht, 2005 for a review; Ohl and Scheich, 2005). While these maps are thought to represent long-term experience with one’s auditory environment (Schreiner and Polley, 2014), they demonstrate high mutability in adults, in that cortical reorganizations may be triggered by task demands as well as the attentional state of the animal (Ahissar et al., 1992, 1998; Fritz et al., 2003, 2010; Fritz J.B. et al., 2005; Polley et al., 2006; for a review see Jääskeläinen and Ahveninen, 2014). In fact, plasticity is not observed when the stimuli are not behaviorally relevant for the organism (Ahissar et al., 1992; Polley et al., 2006; Fritz et al., 2010). Behaviorally relevant experience with a set of tones is known to lead to rapid tonotopic map expansion (Recanzone et al., 1993; Polley et al., 2006; Bieszczad and Weinberger, 2010), sharper receptive field tunings (Recanzone et al., 1993), and greater neuronal synchrony (Kilgard et al., 2007). Notably, these changes appear to have a direct effect on subsequent performance wherein larger cortical map expansion and sharper receptive field tunings are associated with greater improvements in performance following training (Recanzone, 2003). Further, the changes in spectro-temporal receptive field selectivity and inhibition persist for hours after learning, even during subsequent passive listening (Fritz et al., 2003). More recent work by Reed et al. (2011) suggests that while cortical map expansion may be triggered by perceptual learning, these states do not need to be maintained in order to preserve perceptual performance gains. They argue that the function of cortical map expansions is to identify the most efficient circuitry to support a behaviorally relevant, perceptual improvement. Once efficient circuitry is established, the system is able to preserve enhancement in performance via the discovered circuitry despite any subsequent retraction in cortical map representation.

Beyond tonotopic changes, other modes of plasticity in auditory cortex have been found as a consequence of auditory training. For example, experience discriminating spectrally structured auditory gratings (often referred to as auditory spectral ripples) leads to significant changes in the spectral and spectro-temporal receptive field bandwidth of neurons in auditory cortex (Keeling et al., 2008; Yin et al., 2014). These changes, if present in humans, would provide a mechanism that supports the perceptual adaptation to complex sounds, such as phonemes or chord classification (e.g., Schreiner and Calhoun, 1994; Kowalski et al., 1995; Keeling et al., 2008). Besides changes in spectral bandwidth receptivity, auditory training in adult animals can fully correct atypical temporal processing found in auditory cortex due to long-term auditory deprivation, such that normal following capacity and spike-timing precision are found after training (Beitel et al., 2003; Zhou et al., 2012). Crucially, training also appears to induce object-based or category-level processing, in that behaviorally relevant experience engenders complex, categorical representations that go beyond acoustic feature processing (King and Nelken, 2009; Bathellier et al., 2012; Bao et al., 2013; Lu et al., 2017). More specifically, recent work by Bao et al. (2013) has shown that early training leads to neural selectivity for complex spectral features in that trained sounds show greater population level activation relative to untrained sound. Further, while experienced sounds post-training show a reduction in the number of responding neurons, these elicited responses are greater in magnitude. Importantly, the mechanisms guiding plasticity appear to maintain homeostasis within individual receptive fields, in that inhibitory and excitatory synaptic modifications are coordinated such that they collectively sum to zero across a single neuron’s receptive field (Froemke et al., 2013). Coordination between inhibitory and excitatory modifications within a receptive field are necessary, as changes in long-term potentiation or long-term depression alone would create destabilized network activity that is either hyper or hypo-receptive (Abbott and Nelson, 2000). Importantly, the balancing of synaptic modification within individual receptive fields is predicted by cognitive theories of selective attention, which suggest that while directed attention perceptually boosts salient or behaviorally relevant stimuli, it does so at the expense of other stimuli (for a review see, Treisman, 1969).

Neural evidence for rapid perceptual learning in adults has also been found in humans (for reviews, see Jääskeläinen and Ahveninen, 2014; Lee et al., 2014). Specifically, perceptual training of novel phonetic categories appears to lead to changes in early sensory components of scalp recorded auditory evoked potentials (AEPs), which are thought to arise from auditory cortex (Hari et al., 1980; Wood and Wolpaw, 1982; Näätänen and Picton, 1987), suggesting that experience-contingent, perceptual reorganization similarly occurs in humans (e.g., Tremblay et al., 2001; Reinke et al., 2003; Alain et al., 2007, 2015; Ben-David et al., 2011). A recent fMRI and AEP study by de Souza et al. (2013) has shown that rapid perceptual learning is marked not only by a reorganization in sensory cortex but in higher level areas such as left and right superior temporal gyrus and left inferior frontal gyrus. Importantly, their findings suggest that perceptual reorganization due to training is gated by the allocation of attention, implicating behavioral relevance via listening goals as the gating agent in perceptual plasticity. Evidence for this can also be found in the work of Mesgarani and Chang (2012). Using Electrocorticography (ECoG), where electrodes are placed directly on the surface of the brain to record changes in electrical activity from cortex, Mesgarani and Chang (2012) demonstrated that the cortical representations evoked to understand a signal are determined largely by listening goals, such that rapid changes in which talker participants were attending to in multi-talker speech led to immediate changes in population responses in non-primary auditory cortex known to encode critical spectral and temporal features of speech. Specifically, they showed that cortical responses in non-primary auditory cortex are attention-modulated, such that the representations evoked were specific to the talker to which the listener was attending, rather than the external acoustic environment (Mesgarani and Chang, 2012; see also Zion-Golumbic et al., 2013; for review see, Zion-Golumbic and Schroeder, 2012).

As previously mentioned, rapid neural changes in sensory and higher level areas are thought to be the product of the corticofugal system (which includes cortex and subcortical structures such as the inferior colliculus, thalamus, amygdala, hippocampus, and cerebellum), in that bottom-up processes may operate contemporaneously and interactively with top-down driven processes to actively shape signal processing (Suga and Ma, 2003; Slee and David, 2015). Rapid strengthening or diminishing of synapse efficacy can occur within minutes through mechanisms such as long-term potentiation and long-term depression (Cruikshank and Weinberger, 1996; Finnerty et al., 1999; Dinse et al., 2003). As previously mentioned, these alterations appear to be contingent on whether input is behaviorally relevant, especially in the adult animal, suggesting that neural plasticity is gated by top-down or descending systems (Crow, 1968; Kety, 1970; Ahissar et al., 1992; Ahissar et al., 1998; for similar work in adult rats, see Polley et al., 2006) such as the cholinergic and noradrenergic systems that originate from the basal forebrain whose effects are mediated through the regulation of GABA circuits (Ahissar et al., 1996). While there appears to be receptivity in the speech and music community to modeling putatively top-down interactions operating entirely in cortex (George and Hawkins, 2009; Kiebel et al., 2009; Friston, 2010; Moran et al., 2013; Yildiz et al., 2013), very little work has been done to model corticofugal interactions in achieving behaviorally relevant signal processing, as extant neurobiological models of speech and music traditionally limit modeling solely to cortex. As such, the process of perception that extant models puts forth reflects a myopic view of the neural architecture that supports auditory understanding in a world where behavioral relevance is ever-changing (cf. Parvizi, 2009).

Beyond the notion that rapid cortical changes appear to persist for hours, even after the conclusion of a given task (Fritz et al., 2003; Fritz J. et al., 2005; Fritz J.B. et al., 2005), more recent work has started to examine how such rapid changes may be made more robust through other concurrent but more long-term neurobiological mechanisms that may require off-line processing during an inactive period such as sleep (Louie and Wilson, 2001; Brawn et al., 2010). These long-term mechanisms include dendritic remodeling, changes in receptor and transmitter base levels or axonal sprouting or pruning (Sun et al., 2005). Indeed, it is unlikely that immediate changes in cortex are a product of rapid remodeling of synaptic connections, or dendritic expansion or formation, which are likely components of more long-term mechanisms that support learning. Fritz et al. (2013) have suggested that rapid changes in behavior may be driven by changes in the gain of synaptic input onto individual dendritic spines, which may have the necessary architecture to achieve rapid changes. Recent work by Chen et al. (2011) supports this suggestion, as individual synaptic spines on dendrites of layers II to III of A1 neurons in mice are remarkably variable in their tuning frequencies, in that individual neurons possess dendritic spines that are tuned to widely different frequencies, with tunings that are both broad and narrow. As such, the arrangement and pattern of synaptic spines of A1 neurons appears to provides an ideal substrate for rapid cortical receptive field plasticity.

The notion that there are multiple learning mechanisms operating at different time scales concurrently is present in some cognitive learning models (e.g., complementary learning systems, McClelland et al., 1995; Ashby and Maddox, 2005; Ashby et al., 2007). While these models have been important in accounts of learning and memory, they have not been widely incorporated in models of speech and music perception. This omission along with the extreme cortical myopia found within models of speech and music perception reflect an overly simplified, perhaps misguided understanding of the neural mechanisms that underlie perception, as the addition of such mechanisms may drastically alter the processes to be modeled. More explicitly, an important consequence of viewing the perceptual process as highly adaptive is that putatively uninformative variability is no longer something for the system to overcome, but part of the information the system uses to grants perceptual constancy. In this way, it may be our ability to adapt to variable experiences that allows one to assign behaviorally relevant meaning and achieve perceptual stability.

A somewhat different approach to understanding perceptual representations and learning, however, can be found in neural dynamical system models (Laurent et al., 2001; Rabinovich et al., 2001). These models treat a given interpretation for an object as one of many paths through a multidimensional feature space in service of a given listening goal. In essence, the patterns of neural activity in these kinds of systems can form stable trajectories (reflecting different classifications) that are distinct but mutable with experience. These models do not have “stored memories” separate from the processing activity itself within neural populations, so that auditory objects would be represented by the pattern of neural activity over time within the processing network, with different spectro-temporal patterns having different stabilities. This is entirely consistent with Walter Freeman’s work on brain oscillations showing that after rabbits learn a set of odor objects, learning a new odor subsequently alters oscillatory patterns associated with *all* previously learned odors (Freeman, 1978). These types of models do not require a separate stable “representation” for a given object such that different neurons or different network subparts are disjunctively representative of different objects, but instead dynamically create a percept from stable patterns of neural activity arising from the interaction with neural populations. Given that this marks a theoretical shift in ideas about perceptual representation from a traditional neuron doctrine (Barlow, 1972) or cell assembly idea (e.g., Hebb, 1949) in which specific neurons are identified with psychologically distinct objects to the idea that these representations emerge in the patterns of neural activity within a network (see Yuste, 2015), it is unclear how such a framework may be applied to the neural receptive field tuning data just reviewed. One possibility is that changes in behaviorally relevance or training via exposure may shift the activity pattern in a population of neurons from one stable trajectory to another and that mechanisms such as cortical magnification may allow for the most efficient pattern to be found (see, Reed et al., 2011). Models of this sort may provide a different way of conceptualizing short-term and long-term changes in tunings by unifying the impact of experience, not on the formation of representations in memory, but through the dynamic interaction of neural population responses that are sensitive to changes in attention and context.

## Reliance on Recent Experience and Expectations

The evidence cited earlier that receptive fields change as a result of behaviorally relevant experience and that such changes persist after learning, highlights that perceptual constancy may indeed arise through a categorization process that results in attenuation of goal-irrelevant acoustic variability in service of current listening goals. However, such variability may be preserved outside of the veil of perceptual constancy and be incorporated, if lawful, into the representations that guide perception (Elman and McClelland, 1986). Indeed, individuals are faced with continual changes in how phonetic categories are acoustically realized over time at both a community level (Watson et al., 2000; Labov, 2001) and at an idiosyncratic level (Bauer, 1985; Evans and Iverson, 2007). As such, neural representations must preserve aspects of variability outside of processes that produce forms of perceptual constancy.

Work by Tuller et al. (1994), Case et al. (1995) have put forth a non-linear dynamic model of speech perception. In their model, perception is viewed as a dynamical process that is highly context-dependent, such that perceptual constancy is achieved via attraction to “perceptual magnets” that are modified non-linearly through experience. Crucial to their model, listeners remain sensitive to the fine-grain acoustic properties of auditory input as recent experience can induce a shift in perception. Similar to Tuller et al. (1994), Kleinschmidt and Jaeger (2015) have proposed a highly context-dependent model of speech perception. In their model, perceptual stability in speech is achieved through recognition “strategies” that vary depending on the degree to which a signal is familiar based on past experience. This flexible strategic approach based on prior familiarity is critical for successful perception, as a system that is rigidly fixed in acoustic-to-meaning mappings would fail to recognize (perhaps by misclassification) perceptual information that was distinct from past experience, whereas a system that is too flexible might require a listener to continually start from scratch. However, from this view, perceptual constancy is not achieved through the activation of a fixed set of features, but through listening expectations based on the statistics of prior experience. In this way, perceptual constancy arising from such a system could be thought of as an emergent property that results from the comparison of prior experience to bottom-up information from (i) the signal and (ii) recent listening experience (i.e., context).

Within a window of recent experience, what kinds of cues convey to a listener that a deviation from expectations has occurred? Listeners must flexibly shift between different situations that may have different underlying statistical distributions (Qian et al., 2012; Zinszer and Weiss, 2013), using contextual cues that signal a change in an underlying statistical structure (Gebhart et al., 2009). One particularly clear and ecologically relevant contextual cue comes from a change in source information – that is, a change in talker for speech, or instrument for music. For example, when participants learn novel words from distributional probabilities of items across two unrelated artificial languages (i.e., that mark words using different distributional probabilities), they only show reliable transfer of learning across both languages when the differences between languages are contextually cued through different talkers (Weiss et al., 2009). This is presumably because without a contextual cue to index the specific language, listeners must rely on the *overall* accrued statistics of their past experience in relation to the sample of language drawn from the current experience, which may be too noisy to be adequately learned or deployed. More recent work has demonstrated that the kind of cueing necessary to parse incoming distributional information into multiple representations can come from temporal cues as well. Gonzales et al. (2015) found that infants could reliably differentiate statistical input from two accents if temporally separated. This suggests that even in the absence of a salient perceptual distinction between two sources of information (e.g., speaker), listeners can nevertheless use other kinds of cues to meaningfully use variable input to form expectations that can constrain recognition. Indeed, work by Pisoni (1993) has demonstrated that listeners track attributes of speech signals that have been traditionally thought to be unimportant to the recognition process (e.g., a speaker’s speaking rate, emotional state, dialect, and gender) but may be useful in forming expectations that guide and constrain the recognition process. To be clear, these results suggest that experience with the different statistics of pattern sets, given a context cue that appropriately identifies the different sets, may subsequently shape the way listeners direct attention to stimulus properties highlighting a possible way in which top down interactions (via cortical or corticofugal means) may reorganize perception.

Work by Magnuson and Nusbaum (2007) has shown that attention and expectations alone may influence the way listeners tune their perception to context. Specifically, they demonstrated that the performance costs typically associated with adjusting to talker variability, were modulated solely by altering the expectations of hearing one or two talkers. In their study, listeners expecting to hear a single talker did not show performance costs in word recognition when listeners were expecting to hear two talkers, even though the acoustic tokens were identical. Related work by Magnuson et al. (1995) showed that this performance cost is still observed when shifting between two familiar talkers. This example of contextual tuning illustrates that top-down expectations, which occur outside of statistical learning, can fundamentally change how talker variability is accommodated in word recognition. This finding is conceptually similar to research by Niedzielski (1999), who demonstrated that vowel classification differed depending on whether listeners thought the vowels were produced by a speaker from Windsor, Ontario or Detroit, Michigan – cities that have different speech patterns but are close in distance. Similarly Johnson et al. (1999) showed that the perception of “androgynous” speech was altered when presented with a male vs. female face. Linking the domains of speech and music, recent work has demonstrated that the pitch of an identical acoustic signal is processed differently depending on whether the signal is interpreted as spoken or sung (Vanden Bosch der Nederlanden et al., 2015).

Kleinschmidt and Jaeger (2015) has offered a computational approach on how such expectations may influence the perception of a signal. Specifically, they posit that until a listener has enough direct experience with a talker, a listener must supplement their observed input with their prior beliefs, which are brought online via expectations. However, this suggests that prior expectations are only necessary until enough direct experience has accrued. Another possibility, supported by Magnuson and Nusbaum (2007), is that prior expectations are able to shape the interpretation of an acoustic pattern, regardless of accrued experience, as most acoustic patterns are non-deterministic (ambiguous). More specifically, Magnuson and Nusbaum (2007) show that when a many-to-many mapping between acoustic cues and their meanings occurs that this requires more cognitive, active processes, such as a change in expectation that may then direct attention to resolve the recognition uncertainty (cf. Heald and Nusbaum, 2014). Taken together, this suggests that auditory perception cannot be a purely passive, bottom-up process, as expectations about the interpretation of a signal clearly alter the nature of how that signal is processed.

If top-down, attention driven effects are vital in auditory processing, then deficits in such processing should be associated with failures in detecting signal embedded in noise (Atiani et al., 2009; Parbery-Clark et al., 2011), poorer discrimination among stimuli with subtle differences (Edeline et al., 1993), and failure in learning new perceptual categories (Garrido et al., 2009). Indeed, recent work by Perrachione et al. (2016) has argued that the neurophysiological dysfunctions found in dyslexic individuals, which include deficits in these behaviors, arises due to a diminished ability to generate robust, top-down perceptual expectations (for a similar argument see also, Ahissar et al., 2006; Jaffe-Dax et al., 2015).

If recent experience and expectations shape perception, it also follows that the ability to learn signal and pattern statistics is not solely sufficient to explain the empirical accounts of rapid perceptual plasticity within auditory object recognition. Changes in expectations appear to alter the priors the observer uses and may do so by violating the local statistics (prior context), such as when a talker changes. Further, there must be some processing by which one may resolve the inherent ambiguity or uncertainty that arises from the fact that the environment can be represented by multiple associations among cues. Listeners must determine the relevant associations weighing the given context under a given listening goal in order to direct attention appropriately (cf. Heald and Nusbaum, 2014). We argue that the uncertainty in weighing potential interpretations puts a particular emphasis on recent experience, as temporally local changes in contextual cues or changes in the variance of the input can signal to a listener that the underlying statistics have changed, altering how attention is distributed among the available cues in order to appropriately interpret a given signal. Importantly, this window of recent experience may also help solidify or alter listener expectations. In this way, recent experience may act as a buffer or an anchor against which the current signal and current representations are compared to previous experience. This would allow for rapid adaptability across a wide range of putatively stable representations, such as note category representations for AP possessors (Hedger et al., 2013), linguistic representations of pitch (Dolscheid et al., 2013), and phonetic category representations (Liberman et al., 1956; Ladefoged and Broadbent, 1957; Mann, 1986; Evans and Iverson, 2004; Huang and Holt, 2012).

It is important to consider exactly how plasticity engendered by a short-term window relates to a putatively stable, long-term representation of an auditory object. Given the behavioral and neural evidence previously discussed, it does not appear to be the case that auditory representations are static entities once established. Instead, auditory representations appear to be heavily influenced by recent perceptual context. Further, these changes persist in time after learning has concluded. However, this does not imply that there is no inherent stability built into the perceptual system. As previously discussed, perceptual categories in speech and music are not freestanding entities, but rather are a part of a constellation of categories that possess meaningful relationships with one another. Stability may exist through interconnections that exist in the category systems. Long-term neural mechanisms may work to remove rapid cortical changes that are inconsistent with the system, while in other cases, allow such changes to generalize to the rest of the system in order to achieve consistency.

## Conclusion

The present paper has addressed the apparent paradox between experiencing perceptual constancy and dynamic perceptual flexibility in auditory object recognition. Two critical factors in this issue are the problem of acoustic variability and the reliance of listeners on recent experience. Specifically, we have argued that the process of achieving plasticity in audition necessarily entails that one must retain the ability to perceive acoustic variance independent of current listening goals. This is because a system that completely attenuates putatively “irrelevant” variance, by definition, has a single representational structure and assesses incoming perceptual information through a fixed lens. This would necessarily prevent individuals from flexibly adapting to behaviorally relevant changes in their environment. This view also suggests that learning is an important part of the recognition process, as listeners must be able to rapidly learn from and adapt to changes in the statistical distributions of their acoustic environments. A goal for future research should be to examine the degree to which perceptual learning is influenced by listening goals and expectations. More specifically, while perceptual constancy may be goal driven, we have argued that perceptual learning may occur to some extent outside of perceptual constancy. In addition to maintaining sensitivity to acoustic variance, we have argued that a reliance on *recent* experience is necessary for individuals to flexibility adapt to changes in their environment. Recent experience provides a window through which the given signal and current representations are compared to previous knowledge, in that it contains meaningful cues as to when one should switch to an alternate sound-to-meaning mapping. Future work should examine the neural and cognitive mechanisms that underlie this process. Further, extant models of speech and music perception should be updated to reflect the importance of variability and short-term experience in the instantiation of both perceptual flexibility and constancy.

## Author Contributions

SH and SVH wrote the first draft of the manuscript. HN provided comments on the draft, and all authors revised the manuscript to its final form.

## Conflict of Interest Statement

The authors declare that the research was conducted in the absence of any commercial or financial relationships that could be construed as a potential conflict of interest.

## Footnotes

1 Although for exceptions, see Tuller et al. (1994), Case et al. (1995), Mirman et al. (2006), Lancia and Winter (2013), and Kleinschmidt and Jaeger (2015).

## References

[B1] Abbott L. F. Nelson S. B. (2000). Synaptic plasticity: taming the beast. *Nat. Neurosci.* 3 1178–1183. 10.1038/81453 11127835

[B2] Ahissar E. Abeles M. Ahissar M. Haidarliu S. Vaadia E. (1998). Hebbian-like functional plasticity in the auditory cortex of the behaving monkey. *Neuropharmacology* 37 633–655. 10.1016/S0028-3908(98)00068-9 9705003

[B3] Ahissar E. Haidarliu S. Shulz D. E. (1996). Possible involvement of neuromodulatory systems in cortical Hebbian-like plasticity. *J. Physiol. Paris* 90 353–360. 10.1016/S0928-4257(97)87919-3 9089513

[B4] Ahissar E. Vaadia E. Ahissar M. Bergman H. Arieli A. Abeles M. (1992). Dependence of cortical plasticity on correlated activity of single neurons and on behavioral context. *Science* 257 1412–1415. 10.1126/science.1529342 1529342

[B5] Ahissar M. Lubin Y. Putter-Katz H. Banai K. (2006). Dyslexia and the failure to form a perceptual anchor. *Nat. Neurosci.* 9 1558–1564. 10.1038/nn1800 17115044

[B6] Alain C. Da Zhu K. He Y. Ross B. (2015). Sleep-dependent neuroplastic changes during auditory perceptual learning. *Neurobiol. Learn. Mem.* 118 133–142. 10.1016/j.nlm.2014.12.001 25490057

[B7] Alain C. Snyder J. S. He Y. Reinke K. S. (2007). Changes in auditory cortex parallel rapid perceptual learning. *Cereb. Cortex* 17 1074–1084. 10.1093/cercor/bhl018 16754653

[B8] Anantharaman J. N. Krishnamurthy A. K. Feth L. L. (1993). Intensity-weighted average of instantaneous frequency as a model for frequency discrimination. *J. Acoust. Soc. Am.* 94 723–729. 10.1121/1.406889 8370877

[B9] Ashby F. G. Ennis J. M. Spiering B. J. (2007). A neurobiological theory of automaticity in perceptual categorization. *Psychol. Rev.* 114 632–656. 10.1037/0033-295x.114.3.632 17638499

[B10] Ashby F. G. Maddox W. T. (2005). Human category learning. *Annu. Rev. Psychol.* 56 149–178. 10.1146/annurev.psych.56.091103.070217 15709932

[B11] Athos E. A. Levinson B. Kistler A. Zemansky J. Bostrom A. Freimer N. (2007). Dichotomy and perceptual distortions in absolute pitch ability. *Proc. Natl. Acad. Sci. U.S.A.* 104 14795–14800. 10.1073/pnas.0703868104 17724340 PMC1959403

[B12] Atiani S. Elhilali M. David S. V. Fritz J. B. Shamma S. A. (2009). Task difficulty and performance induce diverse adaptive patterns in gain and shape of primary auditory cortical receptive fields. *Neuron* 61 467–480. 10.1016/j.neuron.2008.12.027 19217382 PMC3882691

[B13] Bachem A. (1955). Absolute pitch. *J. Acoust. Soc. Am.* 27 1180–1185. 10.1121/1.1908155

[B14] Baharloo S. Johnston P. A. Service S. K. Gitschier J. Freimer N. B. (1998). Absolute pitch: an approach for identification of genetic and nongenetic components. *Am. J. Hum. Genet.* 62 224–231. 10.1086/301704 9463312 PMC1376881

[B15] Bao S. Chang E. F. Teng C. L. Heiser M. A. Merzenich M. M. (2013). Emergent categorical representation of natural, complex sounds resulting from the early post-natal sound environment. *Neuroscience* 248 30–42. 10.1016/j.neuroscience.2013.05.056 23747304 PMC3838508

[B16] Barlow H. B. (1972). Single units and sensation: a neuron doctrine for perceptual psychology? *Perception* 1 371–394. 10.1068/p010371 4377168

[B17] Barrett J. Paus T. (2002). Affect-induced changes in speech production. *Exp. Brain Res.* 146 531–537. 10.1007/s00221-002-1229-z 12355282

[B18] Bathellier B. Ushakova L. Rumpel S. (2012). Discrete neocortical dynamics predict behavioral categorization of sounds. *Neuron* 76 435–449. 10.1016/j.neuron.2012.07.008 23083744

[B19] Bauer L. (1985). Tracing phonetic change in the received pronunciation of British English. *J. Phonet.* 13 61–81.

[B20] Beitel R. E. Schreiner C. E. Cheung S. W. Wang X. Merzenich M. M. (2003). Reward-dependent plasticity in the primary auditory cortex of adult monkeys trained to discriminate temporally modulated signals. *Proc. Natl. Acad. Sci. U.S.A.* 100 11070–11075. 10.1073/pnas.1334187100 12941865 PMC196928

[B21] Ben-David B. M. Campeanu S. Tremblay K. L. Alain C. (2011). Auditory evoked potentials dissociate rapid perceptual learning from task repetition without learning. *Psychophysiology* 48 797–807. 10.1111/j.1469-8986.2010.01139.x 21054432

[B22] Best C. T. McRoberts G. W. Sithole N. M. (1988). Examination of perceptual reorganization for nonnative speech contrasts: zulu click discrimination by English-speaking adults and infants. *J. Exp. Psychol.* 14 345–360. 10.1037/0096-1523.14.3.345 2971765

[B23] Bidelman G. M. Hutka S. Moreno S. (2013). Tone language speakers and musicians share enhanced perceptual and cognitive abilities for musical pitch: evidence for bidirectionality between the domains of language and music. *PLoS ONE* 8:e60676 10.1371/journal.pone.0060676 PMC3614545 23565267

[B24] Bidelman G. M. Weiss M. W. Moreno S. Alain C. (2014). Coordinated plasticity in brainstem and auditory cortex contributes to enhanced categorical speech perception in musicians. *Eur. J. Neurosci.* 40 2662–2673. 10.1111/ejn.12627 24890664

[B25] Bieszczad K. M. Weinberger N. M. (2010). Representational gain in cortical area underlies increase of memory strength. *Proc. Natl. Acad. Sci. U.S.A.* 107 3793–3798. 10.1073/pnas.1000159107 20133679 PMC2840533

[B26] Billman D. Knutson J. F. (1996). Unsupervised concept learning and value systematicity: a complex whole aids learning the parts. *J. Exp. Psychol. Learn. Mem. Cogn.* 22 458–475. 10.1037/0278-7393.22.2.458 8901345

[B27] Bradlow A. R. Pisoni D. B. Akahane-Yamada R. Tohkura Y. I. (1997). Training Japanese listeners to identify English/r/and/l: IV. Some effects of perceptual learning on speech production. *J. Acoust. Soc. Am.* 101 2299–2310. 10.1121/1.418276 9104031 PMC3507383

[B28] Brady P. T. (1970). Fixed-scale mechanism of absolute pitch. *J. Acoust. Soc. Am.* 48 883–887. 10.1121/1.1912227 5480385

[B29] Brawn T. P. Nusbaum H. C. Margoliash D. (2010). Sleep-dependent consolidation of auditory discrimination learning in adult starlings. *J. Neurosci.* 30 609–613. 10.1523/JNEUROSCI.4237-09.2010 20071524 PMC2824332

[B30] Bregman A. S. (1990). *Auditory Scene Analysis: The Perceptual Organization of Sound.* Cambridge, MA: MIT Press.

[B31] Bruner J. S. (1973). *Beyond the Information Given: Studies in the Psychology of Knowing.* New York, NY: Norton.

[B32] Bruner J. Goodnow J. J. Austin G. A. (1956). *A Study of Thinking.* New York, NY: John Wiley & Sons.

[B33] Carney A. E. Widin G. P. Viemeister N. F. (1977). Noncategorical perception of stop consonants differing in VOT. *J. Acoust. Soc. Am.* 62 961–970. 10.1121/1.381590 908791

[B34] Case P. Tuller B. Ding M. Kelso J. A. (1995). Evaluation of a dynamical model of speech perception. *Percept. Psychophys.* 57 977–988. 10.3758/BF03205457 8532501

[B35] Chen X. Leischner U. Rochefort N. L. Nelken I. Konnerth A. (2011). Functional mapping of single spines in cortical neurons in vivo. *Nature* 475 501–505. 10.1038/nature10193 21706031

[B36] Cherry E. C. (1953). Some experiments on the recognition of speech, with one and with two ears. *J. Acoust. Soc. Am.* 25 975–979. 10.1121/1.1907229

[B37] Chomsky N. Halle M. (1968). *The Sound Pattern of ENGLISH.* New York, NY: Harper & Row.

[B38] Clarke C. Luce P. (2005). “Perceptual adaptation to speaker characteristics: VOT boundaries in stop voicing categorization,” in *Proceedings of the ISCA Workshop on Plasticity in Speech Perception*, London.

[B39] Clayards M. Tanenhaus M. K. Aslin R. N. Jacobs R. A. (2008). Perception of speech reflects optimal use of probabilistic speech cues. *Cognition* 108 804–809. 10.1016/j.cognition.2008.04.004 18582855 PMC2582186

[B40] Cooper W. (1974). Adaptation of phonetic feature analyzers for place of articulation. *J. Acoust. Soc. Am.* 56 617–627. 10.1121/1.1903300 4411861

[B41] Crow T. J. (1968). Cortical synapses and reinforcement: a hypothesis. *Nature* 219 736–737. 10.1038/219736a0 5667068

[B42] Crozier J. B. (1997). Absolute pitch: practice makes perfect, the earlier the better. *Psychol. Music* 25 110–119. 10.1177/0305735697252002

[B43] Cruikshank S. J. Weinberger N. M. (1996). Receptive-field plasticity in the adult auditory cortex induced by Hebbian covariance. *J. Neurosci.* 16 861–875. 8551366 10.1523/JNEUROSCI.16-02-00861.1996 PMC6578626

[B44] Dahan D. Magnuson J. S. Tanenhaus M. K. Hogan E. (2001). Subcategorical mismatches and the time course of lexical access: evidence for lexical competition. *Lang. Cogn. Process.* 16 507–534. 10.1080/01690960143000074

[B45] de Boer J. Thornton A. R. D. (2008). Neural correlates of perceptual learning in the auditory brainstem: efferent activity predicts and reflects improvement at a speech-in-noise discrimination task. *J. Neurosci.* 28 4929–4937. 10.1523/JNEUROSCI.0902-08.2008 18463246 PMC6670751

[B46] de Souza A. C. S. Yehia H. C. Sato M. A. Callan D. (2013). Brain activity underlying auditory perceptual learning during short period training: simultaneous fMRI and EEG recording. *BMC Neurosci.* 14:8 10.1186/1471-2202-14-8 PMC3557158 23316957

[B47] Deutsch D. (2013). “Absolute pitch,” in *The Psychology of Music*, 3rd Edn, ed. Deutsch D. (San Diego, CA: Elsevier), 141–182. 10.1016/B978-0-12-381460-9.00005-5

[B48] Deutsch D. Henthorn T. Dolson M. (2004). Absolute pitch, speech, and tone language: some experiments and a proposed framework. *Music Percept.* 21 339–356. 10.1525/mp.2004.21.3.339

[B49] Dinse H. R. Ragert P. Pleger B. Schwenkreis P. Tegenthoff M. (2003). Pharmacological modulation of perceptual learning and associated cortical reorganization. *Science* 301 91–94. 10.1126/science.1085423 12843392

[B50] Dohn A. Garza-Villarreal E. A. Ribe L. R. Wallentin M. Vuust P. (2014). Musical activity tunes up absolute pitch ability. *Music Perception* 31 359–371. 10.1525/mp.2014.31.4.359

[B51] Dolscheid S. Shayan S. Majid A. Casasanto D. (2013). The thickness of musical pitch: psychophysical evidence for linguistic relativity. *Psychol Sci.* 24 613–621. 10.1177/0956797612457374 23538914

[B52] Edeline J. M. Pham P. Weinberger N. M. (1993). Rapid development of learning-induced receptive field plasticity in the auditory cortex. *Behav. Neurosci.* 107 539–551. 10.1037/0735-7044.107.4.539 8397859

[B53] Elman J. L. McClelland J. L. (1986). “Exploiting lawful variability in the speech wave,” in *Invariance and Variability in Speech Processes*, eds Perkell J. S. Klatt D. H. (Hillsdale, NJ: Lawrence Erlbaum Associates, Inc), 360–385.

[B54] Evans B. G. Iverson P. (2004). Vowel normalization for accent: an investigation of best exemplar locations in northern and southern British English sentences. *J. Acoust. Soc. Am.* 115 352–361. 10.1121/1.1635413 14759027

[B55] Evans B. G. Iverson P. (2007). Plasticity in vowel perception and production: a study of accent change in young adults. *J. Acoust. Soc. Am.* 121 3814–3826. 10.1121/1.2722209 17552729

[B56] Fant G. (1960). *Acoustic Theory of Speech Production*, 2nd Edn. The Hague: Mouton.

[B57] Feldman D. E. Brecht M. (2005). Map plasticity in somatosensory cortex. *Science* 310 810–815. 10.1126/science.1115807 16272113

[B58] Feldman N. H. Griffiths T. L. Goldwater S. Morgan J. L. (2013). A role for the developing lexicon in phonetic category acquisition. *Psychol. Rev.* 120 751–778. 10.1037/a0034245 24219848 PMC3873724

[B59] Fenn K. M. Nusbaum H. C. Margoliash D. (2003). Consolidation during sleep of perceptual learning of spoken language. *Nature* 425 614–616. 10.1038/nature01951 14534586

[B60] Fenn K. M. Shintel H. Atkins A. S. Skipper J. I. Bond V. C. Nusbaum H. C. (2011). When less is heard than meets the ear: change deafness in a telephone conversation. *Q. J. Exp. Psychol.* 64 1442–1456. 10.1080/17470218.2011.570353 21604232

[B61] Finnerty G. T. Roberts L. S. Connors B. W. (1999). Sensory experience modifies the short-term dynamics of neocortical synapses. *Nature* 400 367–371. 10.1038/22553 10432115

[B62] Fitch R. H. Miller S. Tallal P. (1997). Neurobiology of speech perception. *Annu. Rev. Neurosci.* 20 331–353. 10.1146/annurev.neuro.20.1.331 9056717

[B63] Fowler C. A. Galantucci B. (2005). “The relation of speech perception and speech production,” in *The Handbook of Speech Perception*, eds Pisoni D. B. Remez R. E. (Oxford: Blackwell Publishing Ltd).

[B64] Francis A. Nusbaum H. C. (2009). Effects of intelligibility on working memory demand for speech perception. *Attent. Percept. Psychophys.* 71 1360–1374. 10.3758/APP.71.6.1360 19633351

[B65] Francis A. L. Nusbaum H. C. (2002). Selective attention and the acquisition of new phonetic categories. *J. Exp. Psychol. Hum. Percept. Perform.* 28 349–366. 10.1037/0096-1523.28.2.349 11999859

[B66] Francis A. L. Nusbaum H. C. Fenn K. (2007). Effects of training on the acoustic–phonetic representation of synthetic speech. *J. Speech Lang. Hear Res.* 50 1445–1465. 10.1044/1092-4388(2007/100) 18055767

[B67] Freeman W. J. (1978). Spatial properties of an EEG event in the olfactory bulb and cortex. *Electroencephalogr. Clin. Neurophysiol.* 44 586–605. 10.1016/0013-4694(78)90126-8 77765

[B68] Friederici A. D. (2012). The cortical language circuit: from auditory perception to sentence comprehension. *Trends Cogn. Sci.* 16 262–268. 10.1016/j.tics.2012.04.001 22516238

[B69] Friston K. J. (2010). The free-energy principle: a unified brain theory? *Nat. Rev. Neurosci.* 11 127–138. 10.1038/nrn2787 20068583

[B70] Fritz J. Elhilali M. Shamma S. (2005). Active listening: task-dependent plasticity of spectrotemporal receptive fields in primary auditory cortex. *Hear. Res.* 206 159–176. 10.1016/j.heares.2005.01.015 16081006

[B71] Fritz J. Shamma S. Elhilali M. Klein D. (2003). Rapid task-related plasticity of spectrotemporal receptive fields in primary auditory cortex. *Nat. Neurosci.* 6 1216–1223. 10.1038/nn1141 14583754

[B72] Fritz J. B. David S. Shamma S. (2013). “Attention and dynamic, task-related receptive field plasticity in adult auditory cortex,” in *Neural Correlates of Auditory Cognition*, eds Cohen Y. E. Popper A. N. Fay R. R. (New York, NY: Springer), 251–291.

[B73] Fritz J. B. David S. V. Radtke-Schuller S. Yin P. Shamma S. A. (2010). Adaptive, behaviorally gated, persistent encoding of task-relevant auditory information in ferret frontal cortex. *Nat. Neurosci.* 13 1011–1019. 10.1038/nn.2598 20622871 PMC2921886

[B74] Fritz J. B. Elhilali M. Shamma S. A. (2005). Differential dynamic plasticity of A1 receptive fields during multiple spectral tasks. *J. Neurosci.* 25 7623–7635. 10.1523/JNEUROSCI.1318-05.2005 16107649 PMC6725393

[B75] Froemke R. C. Carcea I. Barker A. J. Yuan K. Seybold B. A. Martins A. R. (2013). Long-term modification of cortical synapses improves sensory perception. *Nat. Neurosci.* 16 79–88. 10.1038/nn.3274 23178974 PMC3711827

[B76] Garrido M. I. Kilner J. M. Kiebel S. J. Stephan K. E. Baldeweg T. Friston K. J. (2009). Repetition suppression and plasticity in the human brain. *Neuroimage* 48 269–279. 10.1016/j.neuroimage.2009.06.034 19540921 PMC2821573

[B77] Gay T. (1978). Effect of speaking rate on vowel formant movements. *J. Acoust. Soc. Am.* 63 223–230. 10.1121/1.381717 632415

[B78] Gebhart A. L. Aslin R. N. Newport E. L. (2009). Changing structures in midstream: learning along the statistical garden path. *Cogn. Sci.* 33 1087–1116. 10.1111/j.1551-6709.2009.01041.x 20574548 PMC2889674

[B79] George D. Hawkins J. (2009). Towards a mathematical theory of cortical micro-circuits. *PLoS Comput. Biol.* 5:e1000532 10.1371/journal.pcbi.1000532 PMC2749218 19816557

[B80] Gerstman L. (1968). Classification of self-normalized vowels. *IEEE Trans. Audio Electroacoust.* 16 78–80. 10.1109/TAU.1968.1161953

[B81] Giard M. H. Collet L. Bouchet P. Pernier J. (1994). Auditory selective attention in the human cochlea. *Brain Res.* 633 353–356. 10.1016/0006-8993(94)91561-X 8137171

[B82] Gockel H. Moore B. C. Carlyon R. P. (2001). Influence of rate of change of frequency on the overall pitch of frequency-modulated tones. *J. Acoust. Soc. Am.* 109 701–712. 10.1121/1.1342073 11248974

[B83] Goldstone R. L. (1994). Influences of categorization on perceptual discrimination. *J. Exp. Psychol. Gen.* 123 178–200. 10.1037/0096-3445.123.2.178 8014612

[B84] Goldstone R. L. Hendrickson A. T. (2010). Categorical perception. *Wiley Interdiscipl. Rev. Cogn. Sci.* 1 69–78. 10.1002/wcs.26 26272840

[B85] Goldstone R. L. Kersten A. Cavalho P. F. (2012). “Concepts and categorization,” in *Comprehensive Handbook of Psychology*: *Experimental Psychology* Vol. 4 eds Healy A. F. Proctor R. W. (Hoboken, NJ: Wiley), 607–630.

[B86] Gonzales K. Gerken L. Gómez R. L. (2015). Does hearing two dialects at different times help infants learn dialect-specific rules? *Cognition* 140 60–71. 10.1016/j.cognition.2015.03.015 25880342 PMC4438559

[B87] Gow D. McMurray B. Tanenhaus M. K. (2003). “Eye movements reveal the time course of multiple context effects in the perception of assimilated speech,” in *Poster presented at The 44th Annual Meeting of the Psychonomics Society*, Vancouver, BC.

[B88] Gureckis T. M. Goldstone R. L. (2008). “The effect of the internal structure of categories on perception,” in *Proceedings of the 30th Annual Conference of the Cognitive Science Society*, (Austin, TX: Cognitive Science Society), 1876–1881.

[B89] Handel S. (1989). *Listening: An Introduction to the Perception of Auditory Events.* Cambridge, MA: MIT Press.

[B90] Handel S. (1993). The effect of tempo and tone duration on rhythm discrimination. *Percept. Psychophys.* 54 370–382. 10.3758/BF03205273 8414896

[B91] Hari R. Aittoniemi K. Järvinen M. L. Katila T. Varpula T. (1980). Auditory evoked transient and sustained magnetic fields of the human brain localization of neural generators. *Exp. Brain Res.* 40 237–240. 10.1007/BF00237543 7428878

[B92] Heald S. L. Nusbaum H. C. (2014). Speech perception as an active cognitive process. *Front. Syst. Neurosci.* 8:35 10.3389/fnsys.2014.00035 PMC3956139 24672438

[B93] Hebb D. O. (1949). *The Organization of Behavior.* Hoboken, NJ: Wiley.

[B94] Hedger S. C. Heald S. L. Nusbaum H. C. (2013). Absolute pitch may not be so absolute. *Psychol. Sci.* 24 1496–1502. 10.1177/0956797612473310 23757308

[B95] Hickok G. Poeppel D. (2007). The cortical organization of speech processing. *Nat. Rev. Neurosci.* 8 393–402. 10.1038/nrn2113 17431404

[B96] Holt L. L. (2005). Temporally nonadjacent nonlinguistic sounds affect speech categorization. *Psychol. Sci.* 16 305–312. 10.1111/j.0956-7976.2005.01532.x 15828978

[B97] Holt L. L. Lotto A. J. (2002). Behavioral examinations of the level of auditory processing of speech context effects. *Hear. Res.* 167 156–169. 10.1016/S0378-5955(02)00383-0 12117538

[B98] Holt L. L. Lotto A. J. (2010). Speech perception as categorization. *Attent. Percept. Psychophys.* 72 1218–1227. 10.3758/APP.72.5.1218 PMC2921848 20601702

[B99] Holt L. L. Lotto A. J. Kluender K. R. (2000). Neighboring spectral content influences vowel identification. *J. Acoust. Soc. Am.* 108 710–722. 10.1121/1.429604 10955638

[B100] Huang J. Holt L. L. (2012). Listening for the norm: adaptive coding in speech categorization. *Front. Psychol.* 3:10 10.3389/fpsyg.2012.00010 PMC3272641 22347198

[B101] Ingvalson E. M. Holt L. L. McClelland J. L. (2012). Can native Japanese listeners learn to differentiate/r–l/on the basis of F3 onset frequency? *Biling. Lang. Cogn.* 15 255–274. 10.1017/S1366728911000447 PMC3538861 23308038

[B102] Ingvalson E. M. McClelland J. L. Holt L. L. (2011). Predicting native English-like performance by native Japanese speakers. *J. Phon.* 39 571–584. 10.1016/j.wocn.2011.03.003 22021941 PMC3196605

[B103] Iverson P. Evans B. G. (2007). Plasticity in vowel perception and production: a study of accent change in young adults. *J. Acoust. Soc. Am.* 121 3814–3826. 10.1121/1.2722209 17552729

[B104] Iverson P. Kuhl P. K. (1995). Mapping the perceptual magnet effect for speech using signal detection theory and multidimensional scaling. *J. Acoust. Soc. Am.* 97 553–562. 10.1121/1.412280 7860832

[B105] Jääskeläinen I. P. Ahveninen J. (2014). Auditory-cortex short-term plasticity induced by selective attention. *Neural Plast.* 2014 1–11. 10.1155/2014/216731 PMC3914570 24551458

[B106] Jaffe-Dax S. Raviv O. Jacoby N. Loewenstein Y. Ahissar M. (2015). A computational model of implicit memory captures Dyslexics’ perceptual deficits. *J. Neurosci.* 35 12116–12126. 10.1523/JNEUROSCI.1302-15.2015 26338323 PMC6605302

[B107] Johnson K. Strand E. A. D’Imperio M. (1999). Auditory–visual integration of talker gender in vowel perception. *J. Phon.* 2727 359–384. 10.1006/jpho.1999.0100

[B108] Joos M. (1948). Acoustic phonetics. *Language* 24 1–136. 10.2307/522229

[B109] Keeling M. D. Calhoun B. M. Krüger K. Polley D. B. Schreiner C. E. (2008). Spectral integration plasticity in cat auditory cortex induced by perceptual training. *Exp. Brain Res.* 184 493–509. 10.1007/s00221-007-1115-9 17896103 PMC2474628

[B110] Kety S. (1970). *Neurochemical Aspects of Emotional Behavior.* New York, NY: Academic Press 10.1016/b978-0-12-102850-3.50010-4

[B111] Kiebel S. J. von Kriegstein K. Daunizeau J. Friston K. J. (2009). Recognizing sequences of sequences. *PLoS Comput. Biol.* 5:e1000464 10.1371/journal.pcbi.1000464 PMC2714976 19680429

[B112] Kilgard M. P. Vazquez J. L. Engineer N. D. Pandya P. K. (2007). Experience dependent plasticity alters cortical synchronization. *Hear. Res.* 229 171–179. 10.1016/j.heares.2007.01.005 17317055 PMC2258141

[B113] King A. J. Nelken I. (2009). Unraveling the principles of auditory cortical processing: can we learn from the visual system? *Nat. Neurosci.* 12 698–701. 10.1038/nn.2308 19471268 PMC3657701

[B114] Kleinschmidt D. F. Jaeger T. F. (2015). Robust speech perception: recognize the familiar, generalize to the similar, and adapt to the novel. *Psychol. Rev.* 122 148 10.1037/a0038695 PMC4744792 25844873

[B115] Kluender K. R. Lotto A. J. Holt L. L. Bloedel S. B. (1998). Role of experience for language-specific functional mappings for vowel sounds. *J. Acoust. Soc. Am.* 104 3568–3582. 10.1121/1.423939 9857515

[B116] Kobayashi T. Nisijma K. Ehara Y. Otsuka K. Kato S. (2001). Pitch perception shift: a rare-side effect of carbamazepine. *Psychiatry Clin. Neurosci.* 55 415–417. 10.1046/j.1440-1819.2001.00883.x 11442894

[B117] Kowalski N. Versnel H. Shamma S. A. (1995). Comparison of responses in the anterior and primary auditory fields of the ferret cortex. *J. Neurophysiol.* 73 1513–1523. 7643163 10.1152/jn.1995.73.4.1513

[B118] Krumhansl C. L. Keil F. C. (1982). Acquisition of the hierarchy of tonal functions in music. *Mem. Cogn.* 10 243–251. 10.3758/BF03197636 7121246

[B119] Krumhansl C. L. Kessler E. J. (1982). Tracing the dynamic changes in perceived tonal organization in a spatial representation of musical keys. *Psychol. Rev.* 89 334–368. 10.1037/0033-295X.89.4.334 7134332

[B120] Krumhansl C. L. Shepard R. N. (1979). Quantification of the hierarchy of tonal functions within a diatonic context. *J. Exp. Psychol. Hum. Percept. Perform.* 5:579 10.1037/0096-1523.5.4.579 528960

[B121] Kuhl P. K. Williams K. A. Lacerda F. Stevens K. N. Lindblom B. (1992). Linguistic experience alters phonetic perception in infants by 6 months of age. *Science* 255 606–608. 10.1126/science.1736364 1736364

[B122] Labov W. (2001). *Principles of Linguistic Change: Social Factors*, Vol. 2. Oxford: Blackwell.

[B123] Ladefoged P. Broadbent D. E. (1957). Information conveyed by vowels. *J. Acoust. Soc. Am.* 29 98–104. 10.1121/1.1908694 2525139

[B124] Lancia L. Winter B. (2013). The interaction between competition, learning, and habituation dynamics in speech perception. *Lab. Phonol.* 4 221–257. 10.1515/lp-2013-0009

[B125] Laurent G. Stopfer M. Friedrich R. W. Rabinovich M. I. Volkovskii A. Abarbanel H. D. I. (2001). Odor encoding as an active, dynamical process: experiments, computation, and theory. *Annu. Rev. Neurosci.* 24 263–297. 10.1146/annurev.neuro.24.1.263 11283312

[B126] Lee A. K. Larson E. Maddox R. K. Shinn-Cunningham B. G. (2014). Using neuroimaging to understand the cortical mechanisms of auditory selective attention. *Hear. Res.* 307 111–120. 10.1016/j.heares.2013.06.010 23850664 PMC3844039

[B127] Levitin D. J. Rogers S. E. (2005). Absolute pitch: perception, coding, and controversies. *Trends Cogn. Sci.* 9 26–33. 10.1016/j.tics.2004.11.007 15639438

[B128] Liberman A. M. Cooper F. S. Harris K. S. MacNeilage P. F. Studdert-Kennedy M. (1967). “Some observations on a model for speech perception,” in *Models for the Perception of Speech and Visual Form*, ed. Wathen-Dunn W. (Cambridge. Mass: MIT Press).

[B129] Liberman A. M. Delattre P. C. Gerstman L. J. Cooper F. S. (1956). Tempo of frequency change as a cue for distinguishing classes of speech sounds. *J. Exp. Psychol.* 52 127–137. 10.1037/h0041240 13345983

[B130] Liberman A. M. Harris K. S. Hoffman H. S. Griffith B. C. (1957). The discrimination of speech sounds within and across phoneme boundaries. *J. Exp. Psychol.* 54 358–368. 10.1037/h0044417 13481283

[B131] Liberman A. M. Mattingly I. G. (1985). The motor theory of speech perception revised. *Cognition* 21 1–36. 10.1016/0010-0277(85)90021-6 4075760

[B132] Lieberman P. Crelin E. S. Klatt D. H. (1972). Phonetic ability and related anatomy of the newborn and adult human, neanderthal man, and the chimpanzee. *Am. Anthropol.* 74 287–307. 10.1525/aa.1972.74.3.02a00020

[B133] Lim S. J. Holt L. L. (2011). Learning foreign sounds in an alien world: videogame training improves non-native speech categorization. *Cogn. Sci.* 35 1390–1405. 10.1111/j.1551-6709.2011.01192.x 21827533 PMC3166392

[B134] Lindblom B. (1963). Spectrographic study of vowel reduction. *J. Acoust. Soc. Am.* 35 1773–1781. 10.1121/1.1918816

[B135] Lively S. E. Pisoni D. B. Yamada R. A. Tokhura Y. Yamada T. (1994). Training Japanese listeners to identify English /r/ and /l/. Long-term retention of new phonetic categories. *J. Acoust. Soc. Am.* 96 2076–2087. 10.1121/1.410149 7963022 PMC3518835

[B136] Loui P. Wessel D. (2008). Learning and liking an artificial musical system: effects of set size and repeated exposure. *Music. Sci.* 12 207–230. 10.1177/102986490801200202 20151034 PMC2819428

[B137] Louie K. Wilson M. A. (2001). Temporally structured replay of awake hippocampal ensemble activity during rapid eye movement sleep. *Neuron* 29 145–156. 10.1016/S0896-6273(01)00186-6 11182087

[B138] Lu K. Xu Y. Yin P. Oxenham A. J. Fritz J. B. Shamma S. A. (2017). Temporal coherence structure rapidly shapes neuronal interactions. *Nat. Commun.* 8:13900 10.1038/ncomms13900 PMC5228385 28054545

[B139] Lynch M. P. Eilers R. E. (1991). Children’s perception of native and nonnative musical scales. *Music Percept.* 9 121–132. 10.2307/40286162

[B140] Lynch M. P. Eilers R. E. (1992). A study of perceptual development for musical tuning. *Percept. Psychophys.* 52 599–608. 10.3758/BF03211696 1287565

[B141] Lynch M. P. Eilers R. E. Oller D. K. Urbano R. C. (1990). Innateness, experience, and music perception. *Psychol. Sci.* 1 272–276. 10.1111/j.1467-9280.1990.tb00213.x

[B142] Magnuson J. S. Nusbaum H. C. (2007). Acoustic differences, listener expectations, and the perceptual accommodation of talker variability. *J. Exp. Psychol. Hum. Percept. Perform.* 33 391–409. 10.1037/0096-1523.33.2.391 17469975

[B143] Magnuson J. S. Yamada R. A. Nusbaum H. C. (1995). *The Effects of Talker Variability and Familiarity on mora Perception and Talker Identification.* ATR Human Information Processing Research Laboratories Technical Report TR-H-158. Kyoto: ATR Human Information Processing Research Laboratories.

[B144] Maison S. Micheyl C. Collet L. (2001). Influence of focused auditory attention on cochlear activity in humans. *Psychophysiology* 38 35–40. 10.1111/1469-8986.3810035 11321619

[B145] Mann V. A. (1986). Distinguishing universal and language-dependent levels of speech perception: evidence from Japanese listeners’ perception of English “l” and “r”. *Cognition* 24 169–196. 10.1016/S0010-0277(86)80001-4 3816123

[B146] Maye J. Gerken L. (2000). “Learning phonemes without minimal pairs,” in *Proceedings of the 24th Annual Boston University Conference on Language Development* Vol. 2 eds Howell S. C. Fish S. A. Keith-Lucas T. (Somerville, MA: Somerville, MA), 522–533.

[B147] Maye J. Werker J. F. Gerken L. (2002). Infant sensitivity to distributional information can affect phonetic discrimination. *Cognition* 82 B101–B111. 10.1016/S0010-0277(01)00157-3 11747867

[B148] McClelland J. L. Elman J. L. (1986). The TRACE model of speech perception. *Cogn. Psychol.* 18 1–86. 10.1016/0010-0285(86)90015-0 3753912

[B149] McClelland J. L. McNaughton B. L. O’Reilly R. C. (1995). Why there are complementary learning systems in the hippocampus and neocortex: insights from the successes and failures of connectionist models of learning and memory. *Psychol. Rev.* 102 419–457. 10.1037/0033-295X.102.3.419 7624455

[B150] McGurk H. MacDonald J. (1976). Hearing lips and seeing voices. *Nature* 264 746–748. 10.1038/264746a0 1012311

[B151] McLachlan N. Wilson S. (2010). The central role of recognition in auditory perception: a neurobiological model. *Psychol. Rev.* 117 175–196. 10.1037/a0018063 20063967

[B152] McMurray B. Jongman A. (2011). What information is necessary for speech categorization? Harnessing variability in the speech signal by integrating cues computed relative to expectations. *Psychol. Rev.* 118 219–246. 10.1037/a0022325 21417542 PMC3523696

[B153] McMurray B. Tanenhaus M. K. Aslin R. N. (2002). Gradient effects of within-category phonetic variation on lexical access. *Cognition* 86 B33–B42. 10.1016/S0010-0277(02)00157-9 12435537

[B154] Mesgarani N. Chang E. F. (2012). Selective cortical representation of attended speaker in multi-talker speech perception. *Nature* 485 233–236. 10.1038/nature11020 22522927 PMC3870007

[B155] Miller J. L. Baer T. (1983). Some effects of speaking rate on the production of/b/and/w. *J. Acoust. Soc. Am.* 73 1751–1755. 10.1121/1.389399 6863754

[B156] Mirman D. McClelland J. L. Holt L. L. (2006). An interactive hebbian account of lexically guided tuning of speech perception. *Psychon. Bull. Rev.* 13 958–965. 10.3758/BF03213909 17484419 PMC2291357

[B157] Monahan C. B. (1993). “Parallels between pitch and time and how they go together,” in *Psychology and Music: The Understanding of Melody and Rhythm*, eds Tighe T. J. Dowling W. J. (Hillsdale, NJ: Erlbaum).

[B158] Moon S. J. Lindblom B. (1994). Interaction between duration, context, and speaking style in English stressed vowels. *J. Acoust. Soc. Am.* 96 40–55. 10.1121/1.410492

[B159] Moran R. J. Campo P. Symmonds M. Stephan K. E. Dolan R. J. Friston K. J. (2013). Free energy, precision and learning: the role of cholinergic neuromodulation. *J. Neurosci.* 33 8227–8236. 10.1523/JNEUROSCI.4255-12.2013 23658161 PMC4235126

[B160] Näätänen R. Picton T. (1987). The N1 wave of the human electric and magnetic response to sound: a review and an analysis of the component structure. *Psychophysiology* 24 375–425. 10.1111/j.1469-8986.1987.tb00311.x 3615753

[B161] Niedzielski N. (1999). The effect of social information on the perception of sociolinguistic variables. *J. Lang. Soc. Psychol.* 18 62–85. 10.1177/0261927X99018001005

[B162] Nittrouer S. Lowenstein J. H. (2007). Children’s weighting strategies for word-final stop voicing are not explained by auditory sensitivities. *J. Speech Lang. Hear Res.* 50 58–73. 10.1044/1092-4388(2007/005) 17344548 PMC1994088

[B163] Nittrouer S. Miller M. E. (1997). Predicting developmental shifts in perceptual weighting schemes. *J. Acoust. Soc. Am.* 101 2253–2266. 10.1121/1.418207 9104027

[B164] Nusbaum H. C. Schwab E. C. (1986). The role of attention and active processing in speech perception. *Pattern Recogn. Hum. Mach.* 1 113–157. 10.1016/B978-0-12-631403-8.50009-6

[B165] Nygaard L. C. Sommers M. S. Pisoni D. B. (1995). Effects of stimulus variability on perception and representation of spoken words in memory. *Percept. Psychophys.* 57 989–1001. 10.3758/BF03205458 8532502 PMC3495320

[B166] Ohl F. W. Scheich H. (2005). Learning-induced plasticity in animal and human auditory cortex. *Curr. Opin. Neurobiol.* 15 470–477. 10.1016/j.conb.2005.07.002 16009546

[B167] Parbery-Clark A. Skoe E. Lam C. Kraus N. (2009). Musician enhancement for speech-in-noise. *Ear Hear.* 30 653–661. 10.1097/AUD.0b013e3181b412e9 19734788

[B168] Parbery-Clark A. Strait D. L. Kraus N. (2011). Context-dependent encoding in the auditory brainstem subserves enhanced speech-in-noise perception in musicians. *Neuropsychologia* 49 3338–3345. 10.1016/j.neuropsychologia.2011.08.007 21864552 PMC3445334

[B169] Parvizi J. (2009). Corticocentric myopia: old bias in new cognitive sciences. *Trends Cogn. Sci.* 13 354–359. 10.1016/j.tics.2009.04.008 19595625

[B170] Patel A. D. (2011). Why would musical training benefit the neural encoding of speech? The OPERA hypothesis. *Front. Psychol.* 2:142 10.3389/fpsyg.2011.00142 PMC3128244 21747773

[B171] Perrachione T. K. Del Tufo S. N. Winter R. Murtagh J. Cyr A. Chang P. (2016). Dysfunction of rapid neural adaptation in dyslexia. *Neuron* 92 1383–1397. 10.1016/j.neuron.2016.11.020 28009278 PMC5226639

[B172] Pisoni D. B. (1993). Long-term memory in speech perception: some new findings on talker variability, speaking rate and perceptual learning. *Speech Commun.* 13 109–125. 10.1016/0167-6393(93)90063-Q 21461185 PMC3066018

[B173] Pisoni D. B. Aslin R. N. Perey A. J. Hennessy B. L. (1982). Some effects of laboratory training on identification and discrimination of voicing contrasts in stop consonants. *J. Exp. Psychol. Hum. Percept. Perform.* 8 297 10.1037/0096-1523.8.2.297 PMC3495319 6461723

[B174] Pisoni D. B. Lazarus J. H. (1974). Categorical and noncategorical modes of speech perception along the voicing continuum. *J. Acoust. Soc. Am.* 55 328–333. 10.1121/1.1914506 4821837 PMC3517997

[B175] Pisoni D. B. Tash J. (1974). Reaction times to comparisons within and across phonetic categories. *Percept. Psychophys.* 15 285–290. 10.3758/BF03213946 23226881 PMC3515635

[B176] Polley D. B. Steinberg E. E. Merzenich M. M. (2006). Perceptual learning directs auditory cortical map reorganization through top-down influences. *J. Neurosci.* 26 4970–4982. 10.1523/JNEUROSCI.3771-05.2006 16672673 PMC6674159

[B177] Qian T. Jaeger T. F. Aslin R. N. (2012). Learning to represent a multi-context environment: more than detecting changes. *Front. Psychol.* 3:228 10.3389/fpsyg.2012.00228 PMC3400979 22833727

[B178] Rabinovich M. Volkovskii A. Lecanda P. Huerta R. Abarbanel H. D. I. Laurent G. (2001). Dynamical encoding by networks of competing neuron groups: winnerless competition. *Phys. Rev. Lett.* 87:068102 10.1103/physrevlett.87.068102 11497865

[B179] Rauschecker J. P. Scott S. K. (2009). Maps and streams in the auditory cortex: nonhuman primates illuminate human speech processing. *Nat. Neurosci.* 12 718–724. 10.1038/nn.2331 19471271 PMC2846110

[B180] Recanzone G. A. Schreiner C. E. Merzenich M. M. (1993). Plasticity in the frequency representation of primary auditory cortex following discrimination training in adult owl monkeys. *J. Neurosci.* 13 87–103. 8423485 10.1523/JNEUROSCI.13-01-00087.1993 PMC6576321

[B181] Recanzone G. H. (2003). Auditory influences on visual temporal rate perception. *J. Neurophysiol.* 89 1078–1093. 10.1152/jn.00706.2002 12574482

[B182] Reed A. Riley J. Carraway R. Carrasco A. Perez C. Jakkamsetti V. (2011). Cortical map plasticity improves learning but is not necessary for improved performance. *Neuron* 70 121–131. 10.1016/j.neuron.2011.02.038 21482361

[B183] Reinke K. S. He Y. Wang C. Alain C. (2003). Perceptual learning modulates sensory evoked response during vowel segregation. *Cogn. Brain Res.* 17 781–791. 10.1016/S0926-6410(03)00202-7 14561463

[B184] Ross D. A. Olson I. R. Gore J. C. (2003). Absolute pitch does not depend on early musical training. *Ann. N. Y. Acad. Sci.* 999 522–526. 10.1196/annals.1284.065 14681178

[B185] Rush M. A. (1989). *An Experimental Investigation of the Effectiveness of Training on Absolute Pitch in Adult Musicians.* Doctoral dissertation, The Ohio State University, Columbus, OH.

[B186] Saffran J. R. Aslin R. N. Newport E. L. (1996). Statistical learning by 8-month-old infants. *Science* 274 1926–1928. 10.1126/science.274.5294.1926 8943209

[B187] Saffran J. R. Reeck K. Niebuhr A. Wilson D. (2005). Changing the tune: the structure of the input affects infants’ use of absolute and relative pitch. *Dev. Sci.* 8 1–7. 10.1111/j.1467-7687.2005.00387.x 15647061

[B188] Schellenberg E. G. Trehub S. E. (2003). Good pitch memory is widespread. *Psychol. Sci.* 14 262–266. 10.1111/1467-9280.03432 12741751

[B189] Schreiner C. E. Calhoun B. M. (1994). Spectral envelope coding in cat primary auditory cortex: properties of ripple transfer functions. *Audit. Neurosci.* 1 39–62.

[B190] Schreiner C. E. Polley D. B. (2014). Auditory map plasticity: diversity in causes and consequences. *Curr. Opin. Neurobiol.* 24 143–156. 10.1016/j.conb.2013.11.009 24492090 PMC4206409

[B191] Sergeant D. C. Roche S. (1973). Perceptual shifts in the auditory information processing of young children. *Psychol. Music* 1 39–48. 10.1177/030573567312006

[B192] Shamma S. Fritz J. (2014). Adaptive auditory computations. *Curr. Opin. Neurobiol.* 25 164–168. 10.1016/j.conb.2014.01.011 24525107 PMC3981867

[B193] Siegel J. A. Siegel W. (1977). Categorical perception of tonal intervals: musicians can’t tell sharp from flat. *Percept. Psychophys.* 21 399–407. 10.3758/BF03199493

[B194] Slee S. J. David S. V. (2015). Rapid task-related plasticity of spectrotemporal receptive fields in the auditory midbrain. *J. Neurosci.* 35 13090–13102. 10.1523/JNEUROSCI.1671-15.2015 26400939 PMC4579375

[B195] Soley G. Hannon E. E. (2010). Infants prefer the musical meter of their own culture: a cross-cultural comparison. *Dev. Psychol.* 46 286 10.1037/a0017555 20053025

[B196] Stevens K. (1998). *Acoustic Phonetics.* Cambridge, MA: MIT Press.

[B197] Stevens K. N. Blumstein S. E. (1981). “The search for invariant acoustic correlates of phonetic features,” in *Perspectives on the Study of Speech*, eds Eimas P. D. Miller J. L. (Hillsdale, NJ: Erlbaum), 1–38.

[B198] Strait D. L. Kraus N. Skoe E. Ashley R. (2009). Musical experience promotes subcortical efficiency in processing emotional vocal sounds. *Ann. N. Y. Acad. Sci.* 1169 209–213. 10.1111/j.1749-6632.2009.04864.x 19673783

[B199] Strange W. Jenkins J. J. (1978). Role of linguistic experience in the perception of speech. *Percept. Exp.* 1 125–169. 10.1007/978-1-4684-2619-9_5

[B200] Studdert-Kennedy M. Liberman A. M. Harris K. S. Cooper F. S. (1970). Motor theory of speech perception: a reply to Lane’s critical review. *Psychol. Rev.* 77 234–249. 10.1037/h0029078 5454133

[B201] Suga N. Ma X. (2003). Multiparametric corticofugal modulation and plasticity in the auditory system. *Nat. Rev. Neurosci.* 4 783–794. 10.1038/nrn1222 14523378

[B202] Sun W. Mercado E. III Wang P. Shan X. Lee T. C. Salvi R. J. (2005). Changes in NMDA receptor expression in auditory cortex after learning. *Neurosci. Lett.* 374 63–68. 10.1016/j.neulet.2004.10.032 15631898

[B203] Terhardt E. S. Seewan M. M. (1983). Aural key identification and its relationship to absolute pitch. *Music Percept.* 1 63–83. 10.2307/40285250

[B204] Theusch E. Basu A. Gitschier J. (2009). Genome-wide study of families with absolute pitch reveals linkage to 8q24. 21 and locus heterogeneity. *Am. J. Hum. Genet.* 85 112–119. 10.1016/j.ajhg.2009.06.010 19576568 PMC2706961

[B205] Treisman A. M. (1969). Strategies and models of selective attention. *Psychol. Rev.* 76 282–299. 10.1037/h0027242 4893203

[B206] Tremblay K. Kraus N. McGee T. Ponton C. Otis B. (2001). Central auditory plasticity: changes in the N1-P2 complex after speech-sound training. *Ear Hear.* 22 79–90. 10.1097/00003446-200104000-00001 11324846

[B207] Tuller B. Case P. Ding M. Kelso J. A. S. (1994). The nonlinear dynamics of speech categorization. *J. Exp. Psychol. Hum. Percept. Perform.* 20 3–16. 10.1037/0096-1523.20.1.3 8133223

[B208] Van Hedger S. C. Heald S. L. Huang A. Rutstein B. Nusbaum H. C. (2016). Telling in-tune from out-of-tune: widespread evidence for implicit absolute intonation. *Psychon. Bull. Rev.* 24 1–8. 10.3758/s13423-016-1099-1 27383616

[B209] Van Hedger S. C. Heald S. L. Koch R. Nusbaum H. C. (2015). Auditory working memory predicts individual differences in absolute pitch learning. *Cognition* 140 95–110. 10.1016/j.cognition.2015.03.012 25909580

[B210] Vanden Bosch der Nederlanden C. M. Hannon E. E. Snyder J. S. (2015). Finding the music of speech: Musical knowledge influences pitch processing in speech. *Cognition* 143 135–140. 10.1016/j.cognition.2015.06.015 26151370

[B211] Ward W. D. Burns E. M. (1999). Absolute pitch. *Psychol. Music* 2 265–298. 10.1016/B978-012213564-4/50009-3

[B212] Watson C. I. Maclagan M. Harrington J. (2000). Acoustic evidence for vowel change in New Zealand English. *Lang. Var. Change* 12 51–68. 10.1017/S0954394500121039

[B213] Weinberger N. M. (2004). Specific long-term memory traces in primary auditory cortex. *Nat. Rev. Neurosci.* 5 279–290. 10.1038/nrn1366 15034553 PMC3590000

[B214] Weinberger N. M. (2015). New perspectives on the auditory cortex: learning and memory. *Handbook Clin. Neurol.* 129 117–147. 10.1016/B978-0-444-62630-1.00007-X 25726266

[B215] Weiss D. J. Gerfen C. Mitchel A. D. (2009). Speech segmentation in a simulated bilingual environment: a challenge for statistical learning? *Lang. Learn. Dev.* 5 30–49. 10.1080/15475440802340101 24729760 PMC3981102

[B216] Werker J. F. Polka L. (1993). “The ontogeny and developmental significance of language-specific phonetic perception,” in *Developmental Neurocognition: Speech and Face Processing in the First Year of Life. The Netherlands*, eds Boysson-Bardies B. de Schonen S. de Jusczyk P. MacNeilage P. Morton J. (Dordrecht: Kluwer Academic Publishers B.V). 10.1007/978-94-015-8234-6_23

[B217] Werker J. F. Tees R. C. (1983). Developmental changes across childhood in the perception of non-native speech sounds. *Can. J. Psychol.* 37 278–286. 10.1037/h0080725 6616342

[B218] Werker J. F. Tees R. C. (1984). Cross-language speech perception: evidence for perceptual reorganization during the first year of life. *Infant Behav. Dev.* 7 49–63. 10.1016/S0163-6383(84)80022-3

[B219] Wilson S. J. Lusher D. Martin C. L. Rayner G. McLachlan N. (2012). Intersecting factors lead to absolute pitch acquisition that is maintained in a “fixed do” environment. *Music Percept. Interdiscipl. J.* 29 285–296. 10.1525/mp.2012.29.3.285

[B220] Wong P. C. Chan A. H. Roy A. Margulis E. H. (2011). The bimusical brain is not two monomusical brains in one: evidence from musical affective processing. *J. Cogn. Neurosci.* 23 4082–4093. 10.1162/jocn_a_00105 21812560

[B221] Wong P. C. Roy A. K. Margulis E. H. (2009). Bimusicalism: the implicit dual enculturation of cognitive and affective systems. *Music Percept. An Interdiscipl. J.* 27 81–88. 10.1525/mp.2009.27.2.81 PMC2907111 20657798

[B222] Wong P. C. M. Perrachione T. K. (2007). Learning pitch patterns in lexical identification by native English-speaking adults. *Appl. Psychol.* 28 565–585. 10.1017/S0142716407070312

[B223] Wood C. C. Wolpaw J. R. (1982). Scalp distribution of human auditory evoked potentials. II. Evidence for overlapping sources and involvement of auditory cortex. *Electroencephalogr. Clin. Neurophysiol.* 54 25–38. 10.1016/0013-4694(82)90228-0 6177515

[B224] Wu H. Ma X. Zhang L. Liu Y. Zhang Y. Shu H. (2015). Musical experien ce modulates categorical perception of lexical tones by native Chinese speakers. *Front. Psychol.* 6:436 10.3389/fpsyg.2015.00436 PMC4394639 25918511

[B225] Yildiz I. B. von Kriegstein K. Kiebel S. J. (2013). From birdsong to human speech recognition: bayesian inference on a hierarchy of nonlinear dynamical systems. *PLoS Comput. Biol.* 9:e1003219 10.1371/journal.pcbi.1003219 PMC3772045 24068902

[B226] Yin P. Fritz J. B. Shamma S. A. (2014). Rapid spectrotemporal plasticity in primary auditory cortex during behavior. *J. Neurosci.* 34 4396–4408. 10.1523/JNEUROSCI.2799-13.2014 24647959 PMC3960477

[B227] Yuste R. (2015). From the neuron doctrine to neural networks. *Nat. Rev. Neurosci.* 16 487–497. 10.1038/nrn3962 26152865

[B228] Zatorre R. J. Halpern A. R. (1979). Identification, discrimination, and selective adaptation of simultaneous musical intervals. *J. Acoust. Soc. Am.* 65 S40–S40. 10.3758/bf03204164 523282

[B229] Zhou Y. Mesik L. Sun Y. J. Liang F. Xiao Z. Tao H. W. (2012). Generation of spike latency tuning by thalamocortical circuits in auditory cortex. *J. Neurosci.* 32 9969–9980. 10.1523/JNEUROSCI.1384-12.2012 22815511 PMC3470470

[B230] Zhu M. Chen B. Galvin J. J. Fu Q. J. (2011). Influence of pitch, timbre and timing cues on melodic contour identification with a competing masker (L). *J. Acoust. Soc. Am.* 130 3562–3565. 10.1121/1.3658474 22225012 PMC3253593

[B231] Zinszer B. D. Weiss D. J. (2013). “When to hold and when to fold: detecting structural changes in statistical learning,” in *Proceedings of the Thirty-Fifth Annual Conference of the Cognitive Science Society*, eds Knauff M. Pauen M. Sebanz N. Wachsmuth I. (St. Andrews: University of St Andrews), 3858–3863.

[B232] Zion-Golumbic E. Schroeder C. E. (2012). Attention modulates ‘speech-tracking’ at a cocktail party. *Trends Cogn. Sci.* 16 363–364. 10.1016/j.tics.2012.05.004 22651956 PMC3904732

[B233] Zion-Golumbic E. M. Z. Ding N. Bickel S. Lakatos P. Schevon C. A. McKhann G. M. (2013). Mechanisms underlying selective neuronal tracking of attended speech at a “cocktail party”. *Neuron* 77 980–991. 10.1016/j.neuron.2012.12.037 23473326 PMC3891478

